# Synthesis of an enhanced nanobiocatalyst system from *Aspergillus niger* as single green source

**DOI:** 10.1038/s41598-025-31186-9

**Published:** 2025-12-23

**Authors:** Mohamed G. Radwan, Tarek M. Mohamed, Radwa H. Abou-Saleh, Hamed A. Abosharaf

**Affiliations:** 1 Health Sector, Faculty of Science, Galala University, Suez, 43511 Egypt; 2 Biochemistry Division, Chemistry Department, Faculty of Science, Tanta University, Tanta, 31527 Egypt; 3 Nanoscience and technology, Physics Department, Faculty of Science, Galala University, Suez, 43511 Egypt; 4 Biophysics division, Physics Department, Faculty of Science, Mansoura University, Mansoura, 35516 Egypt

**Keywords:** Aspergillus niger, Biogenic iron oxide nanoparticles, Lipase, Nanobiocatalyst, Immobilization, Single-source synthesis, Biochemistry, Biological techniques, Biotechnology, Microbiology, Nanoscience and technology

## Abstract

The development of sustainable nanobiocatalysts is a focal challenge in green chemistry, requiring robust and eco-friendly production methods. This study introduces a single-source strategy that addresses this challenge. By utilizing the fungus *Aspergillus niger* as a single biological factory to simultaneously produce a lipase enzyme and biosynthesize the iron oxide nanoparticles (IONPs) that serve as lipase matrix support. This integrated approach ensures a high degree of compatibility between the enzyme and its nanoparticle support, which was confirmed during the immobilization step by an 81.73% yield and a remarkable 97.4% activity retention. The resulting nanobiocatalyst demonstrated a distinct operational stability, broad pH tolerance, and maintained over 80% of its activity after eight consecutive reuse cycles. In practical applications, the catalyst showed powerful bioremediation capabilities, achieving near-complete (> 95%) removal of industrial dyes and effective oil-stain removal from fabric. Our findings could establish that using a single biological source for both the enzyme and its immobilization matrix offers a new benchmark for future enzyme immobilization technologies.

**Supplementary Information:**

The online version contains supplementary material available at 10.1038/s41598-025-31186-9.

## Introduction

The synergy between nanomaterials and enzymes in nanobiocatalysis holds significant promise for advancing industrial processes^1^. Lipases play a pivotal role in triglyceride hydrolysis^2^ and are widely used in the food, detergent, pharmaceutical, and cosmetic industries. They are also central to biotransformation and sustainable biotechnology initiatives. The growing demand for environmentally friendly biocatalysts underscores their economic and ecological importance^3^. However, the industrial application of lipases is often constrained by challenges related to stability and recovery.

Immobilization on solid supports has emerged as a solution to these limitations by enhancing performance, simplifying recovery, and enabling enzyme reuse^4^. A range of support materials, such as hydrophobic materials, ceramics, and biopolymers, are available^5,6^. Among these, magnetic nanomaterials, specifically iron oxide nanoparticles (IONPs), are efficient for biocatalyst separation^4^. Hematite (α-Fe2O3) IONPs are particularly promising because of their stability, biocompatibility, high surface area, and potential for improved catalytic performance^7,8^. However, traditional IONP synthesis methods often involve high-energy usage, toxic chemicals, and complex purification processes, which limit their scalability and sustainability^9^.

To overcome these challenges, biosynthetic methods, especially those employing fungi, are gaining popularity. These methods offer cost-effective, nontoxic, and biocompatible alternatives for IONP production^10^. Fungi have several advantages, including rapid growth, simple nutrient requirements, ease of handling, and the production of extracellular enzymes and biomolecules essential for metal ion reduction and nanoparticle stabilization^11^. The extracellular production process also simplifies downstream processing^12^ with biomolecules, such as proteins, NADH-dependent reductases, and reducing sugars, which play critical roles in biosynthesis. This fungus-mediated approach provides precise control over nanoparticle size, shape, and crystallinity, which directly affects their functionality and biocompatibility^13^.

In parallel, advanced strategies have increasingly focused on the co-immobilization of multiple, distinct enzymes into a single support to create multifunctional biocatalysts. A key example is the use of hydrogel nanofibers to co-immobilize different enzymes for cascade reactions, as demonstrated by Li et al. and Liang et al.^14,15^. While powerful, these multi-enzyme systems still rely on the conventional multi-source paradigm of combining pre-made components.

**Fig. 1:**
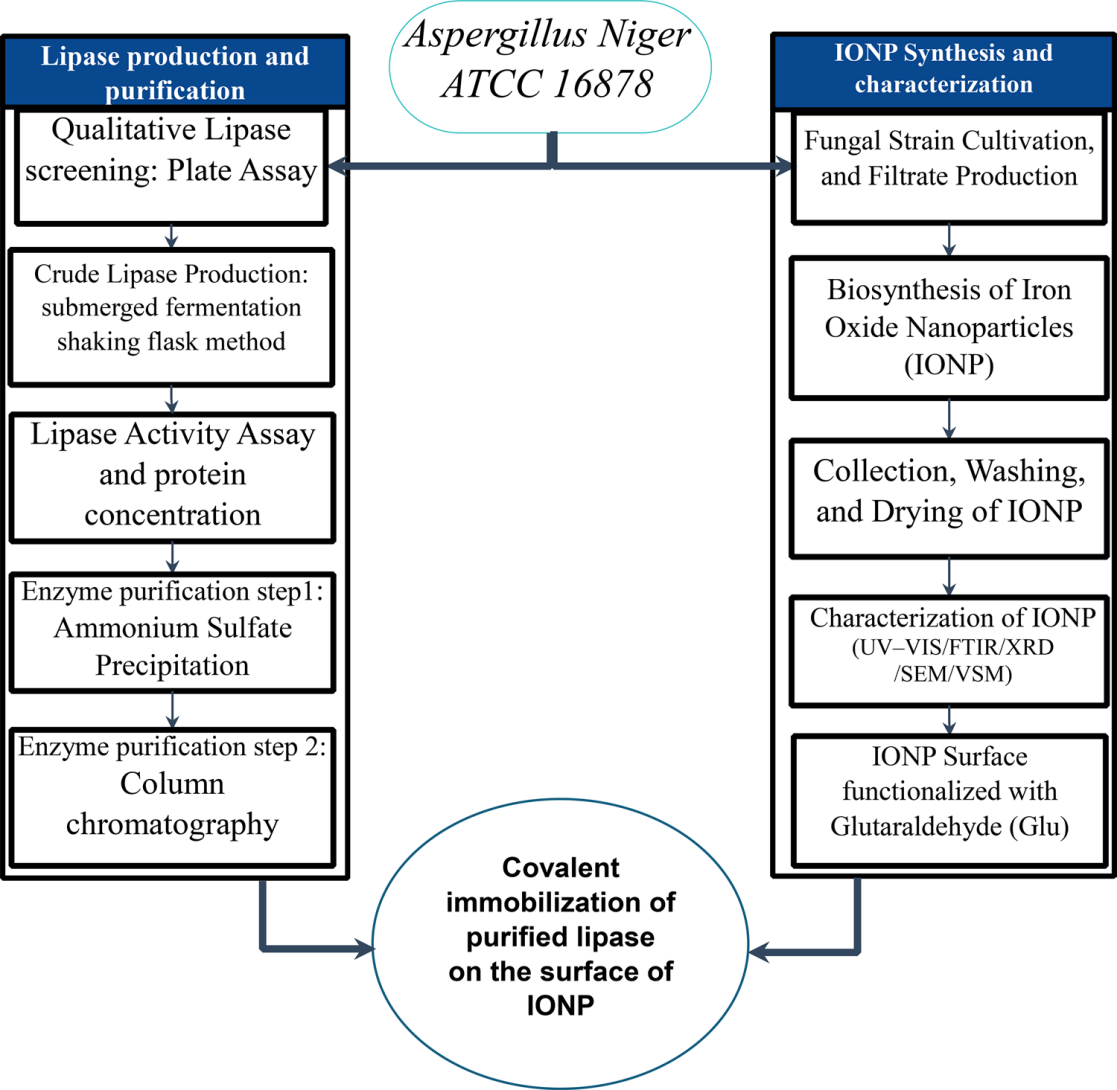
The integrated workflow for single-source nanobiocatalyst synthesis. Aspergillus niger serves as a single source for two parallel pathways: the production of lipase and the green synthesis of iron oxide nanoparticles (IONPs). The two components are then unified via covalent immobilization, a strategy designed to enhance compatibility between the enzyme and its biogenic support, leading to a more stable and effective nanobiocatalyst.

Current approaches to nanobiocatalyst development typically involve immobilizing a purified enzyme from one source onto nanoparticles synthesized via a separate route. For example, studies have reported immobilizing porcine pancreas lipase (lipase L1), Candida rugosa lipase (li-pase L2), and Pseudomonas cepacia lipase (lipase L3) onto chemically synthesized Fe_3_O_4_ nanoparticle^16^, or enzymes from *Candida rugosa* (CRL)onto Multi-walled carbon nanotubes^17^. This conventional, multi-source method, exemplified by numerous studies (see Supplementary Table S1), can lead to suboptimal performance due to biocompatibility issues between the enzyme and the foreign support material^18^. To address this fundamental challenge, we hypothesized that a ‘single-source’ strategy, where the same organism acts as a bio-factory for both the enzyme and the nanoparticle support, would create a more compatible and effective system. Here, we propose and demonstrate a novel, unified workflow using *Aspergillus niger* to simultaneously produce lipase and biosynthesize the iron oxide nanoparticles (IONPs) for its immobilization. The objectives of this study were to develop this integrated system, fully characterize its components, and evaluate the performance of the resulting nanobiocatalyst in challenging applications. This unified workflow, which forms the central concept of our study, is illustrated in Fig. 1.

## Materials and methods

### Materials

P-nitrophenyl palmitate (PNPP) was sourced from Thermo Fisher Scientific, Inc. (USA), and used as the lipase substrate. Peptone water and yeast extract were obtained from HIMEDIA, India, and Sephadex-G-100 was supplied by Solarbio, China. Iron (III) chloride hexahydrate (FeCl₃·6 H₂O), iron (II) sulfate heptahydrate (FeSO₄·7 H₂O), and Tween 20 were purchased from Algomhoria Chemicals, Egypt. The fungal strain *Aspergillus niger* ATCC 16,878 (*A. niger*) was acquired from the Center of Fermentation Biotechnology & Applied Microbiology, Al-Azhar University, Egypt, and deposited in GenBank under the accession number ATCC 16,878.

### Methods

#### Aspergillus Niger lipase production and purification

##### Qualitative screening for lipolytic activity

The lipolytic activity of *A. niger* was determined via a qualitative methyl red plate assay following the protocol described by Samad et al.^19^. The agar plates comprised (w/v) agar (1.5%), peptone (1.5%), CaCl₂ (0.1%), NaCl (0.5%), and Tween 20, with methyl red (0.01%) as the pH indicator. A Tween 20 emulsion was prepared by dissolving 5 g of Arabic gum in 50 mL of distilled water, followed by the addition of 5 mL of Tween 20, stirring, and homogenization. This emulsion was aseptically incorporated into sterile agar medium and cooled to 50 °C. After mixing, *A. niger* was spot inoculated onto the plates, which were subsequently incubated at 28 °C for six days. Lipase activity was visualized as a red halo around the fungal colonies due to the decrease in pH^19^.

##### Submerged fermentation for lipase production

Lipase was produced by the submerged fermentation of *A. niger* in shaking flasks. The broth medium contained (w/v) yeast extract (0.5 g%), peptone (2 g%), NaCl (0.5 g%), Na₂CO₃ (2.5 mg%), and an olive oil emulsion (1.5%). The pH of the medium was adjusted to 7.0, and the medium was sterilized. The medium was aliquoted into Erlenmeyer flasks at a 1:4 (v/v) medium: flask ratio to ensure optimal aeration. Each flask was inoculated with 0.3 mL of an *A. niger* ATCC spore suspension (8 × 10⁷ spores/mL), which was prepared via a hemocytometer. The flasks were incubated at 28 °C with shaking at 120 rpm for six days, which was previously determined to be the optimal duration for lipase production^20^. Crude lipase was harvested by centrifugation at 10,000 ×g for 20 min at 4 °C, and the supernatant was subjected to activity analysis.

##### Lipase activity and protein concentration assays

Lipase activity was measured spectrophotometrically with 4-nitrophenyl palmitate (PNPP) as the substrate according to the method described by Gupta et al.. with slight modifications^21^. The assay, with a total volume of 2 mL, consisted of 790 µM PNPP substrate (4% v/v), 2% v/v Triton X-100, and 1 mL of 0.1 M potassium phosphate buffer (pH 7.5). After a 30-minute incubation at 37 °C, the absorbance of the released p-nitrophenol was measured at 410 nm against a blank. One unit (U) of lipase activity was defined as the amount of enzyme that released 1 µmol of p-nitrophenol per mL of enzyme. The molar extinction coefficient of p-nitrophenol (p-NP) was calculated as 1.27 × 10³ M⁻¹ cm⁻¹ under assay conditions^22^. The protein concentration was determined using the Folin‒Ciocalteu method^23^, and the specific activity was expressed as units per milligram of protein (U/mg protein), which represented the µmol of p-nitrophenol released per mg protein.

#### Partial purification of lipase

Crude lipase was partially purified by ammonium sulfate precipitation at saturation levels ranging from 20% to 80%. The fractions exhibiting the highest lipase activity were selected and subjected to dialysis to remove residual ammonium sulfate. Dialysis was performed overnight at 4 °C using a Visking dialysis membrane (size 3–20/32, Medicell Membranes Ltd., UK) with four buffer changes^24^.

Subsequently, further purification was conducted via a Sephadex G-100 column (60 × 2.25 cm^2^) equilibrated with 0.1 M phosphate buffer (pH 7.5)^25,26^. Fractions (3 mL each) were eluted at intervals of 3 min, and the protein content was determined spectrophotometrically by measuring the absorbance at 280 nm^27^. Fractions with significant absorbance were analyzed for lipase activity using the pNPP assay.

#### Biosynthesis of biogenic iron oxide nanoparticles (IONPs)

Iron oxide nanoparticles (IONPs) were synthesized using our previously published green synthesis method^28^, where iron salt precursors reacts with A. niger filtrate (as a bio reductant and capping agent) under controlled pH and temperature conditions. Briefly, *A. niger* filtrate reacted with $\:{FeCl}_{3}.\:{(H}_{\:2}O)$ (6 mM, final concentration) and $\:{FeSO}_{4}.7\left({H}_{2}O\right)$ (final concentration of 3 mM) at pH 9.0 and 40 °C for 24 h. Nanoparticle formation was visually confirmed from instant color change to brownish. The synthesized IONPs were subsequently purified and dried as detailed in^28^.

### Preparation of the single-source nanobiocatalyst (IONP@Lipase)

Iron oxide nanoparticles (IONPs) were prepared at a concentration of 5 mg/mL in 0.1 M phosphate buffer (pH 7.5), sonicated for 10 min, and activated with 1.0 M glutaraldehyde for 1 h at 25 °C with shaking at 250 rpm. Excess glutaraldehyde was removed by washing with deionized water. Activated nanoparticles (IONP@GLU, 5 mg/mL) were then mixed with purified lipase to achieve a final activity of 71.1 IU in a 3 mL reaction volume. The mixture was then incubated at 4 °C on a shaker at 150 rpm for 5 h. After immobilization, the conjugates were washed three times with sodium chloride solutions of decreasing concentrations (1.0, 0.5, and 0.3 M) to eliminate weakly bound enzymes. The unbound enzyme was discarded, and the IONP@GLU-Lipase conjugates were characterized by biochemical and biophysical characterization. The immobilization yield (IY) and recovered activity (Atr) were calculated via formulas adapted from a previously established method^29^, where:

*IY* (%) = [Total initial enzyme activity (U) – Remaining enzyme units in the supernatant after completing immobilization (U)/Total initial enzyme activity (U)] × 100.

Atr (%) = [Total initial enzyme activity (U)/Actual immobilized enzyme units (U)] × 100.

### Characterization methods

#### Characterization of biosynthesized ionps

Characterization of the synthesized IONPs was performed using UV-Vis spectroscopy, Fourier Transform Infrared Spectroscopy (FTIR), X-ray Diffraction (XRD), Scanning Electron Microscopy (SEM), and Vibrating Sample Magnetometry (VSM), following the procedures reported in our previous work^28^.

#### Characterization of the immobilized nanobiocatalyst (IONP@Lipase)

##### FTIR spectroscopy analysis

Fourier transform infrared (FTIR) spectroscopy was employed to analyze the iron oxide nanoparticles (IONPs) after glutaraldehyde treatment as a surface-functionalizing agent, followed by lipase immobilization. This analysis aimed to detect changes in the surface functional groups resulting from the covalent attachment of the lipase. The FTIR spectra were recorded within the range of 400–4000 cm⁻¹ using a NICOLET iS50 FT-IR spectrometer (Thermo Scientific, USA).

##### Enzyme kinetics and effect of pH, temperature, reusability and storage conditions (Free vs. Immobilized Lipase)

The kinetic parameters of both free and immobilized *A. niger* lipases were analyzed using pNPP as a substrate at concentrations ranging from 0.1 to 1 mM. The Michaelis‒Menten equation was applied to calculate the km and Vmax values via nonlinear regression using Origin2024b software^30^. The effect of both pH and temperature on the activity of free and immobilized lipase was determined. The pH profile was assessed across a range of 4.0–9.0 at 37 °C using standard buffers: acetate (pH 4.0–5.5.0.5), potassium phosphate (pH 6.0–7.5.0.5), and Tris-HCl (pH 8.0–8.5.0.5)^31^. The temperature profile was studied from 20 °C to 50 °C at the optimal pH of 7.5. For these comparative analyses, the activity of each enzyme form (free and immobilized) at its own optimal condition (pH 7.5 and 37 °C, respectively) was defined as 100%. All other activity measurements were then calculated as a percentage relative to this maximum for that specific enzyme form^32^.

The reusability of the immobilized lipase was tested over repeated pNPP assay cycles at pH 7.5 and 37 °C. After each cycle, the nanobiocatalyst was recovered from the reaction mixture by applying an external magnet, washed with buffer to remove any residual substrate or product, and then resuspended in a fresh assay solution for the subsequent cycle. Stability of the free and immobilized enzymes was assayed at different temperatures by incubating samples from both enzymes at different temperatures (4 and 25 °C). Activity was assayed via the standard pNPP colorimetric method for the entire incubation period (90 days), and the IONP-lipase conjugates were magnetically recovered, washed, and resuspended for subsequent assays^33,34^.

### Application studies of the nanobiocatalyst

#### Dye degradation assays (Methyl orange and methylene blue)

MO and MB degradation was assessed using free and immobilized *Aspergillus niger* lipase, alongside a no-enzyme control. All reactions used 50 U of lipase with 15 mg/L dye. Degradation occurred over 6 h, with hourly sample collection and 10-fold dilution. The absorbance was measured spectrophotometrically at 465 nm (MO) and 668 nm (MB). Dye concentrations were determined via preestablished standard curves (0.5–2.5 mg/L).

Dye removal efficiency (%) = [(initial dye concentration – final dye concentration)/initial dye concentration] × 100.

Average deceleration rate (mg/L/h) = [Initial Dye Concentration – Final Dye Concentration]/Time change.

#### Evaluation of oil stain removal from fabric

The defatted (chloroform-boiled) cotton fabric (2 cm × 3 cm) was stained with frying oil and dried (60 °C for 10 min). Seven triplicate groups were tested: (1) dry control, (2) water control (100 mL), (3) 1% heat-inactivated detergent (100 mL), (4) free lipase (40 U/mL, 100 mL), (5) immobilized lipase (40 U, 100 mL), (6) 1% detergent + free lipase (40 U/mL, 100 mL), and (7) 1% detergent + immobilized lipase (40 U, 100 mL).

## Results

### Aspergillus Niger lipase production and purification

**Fig. 2:**
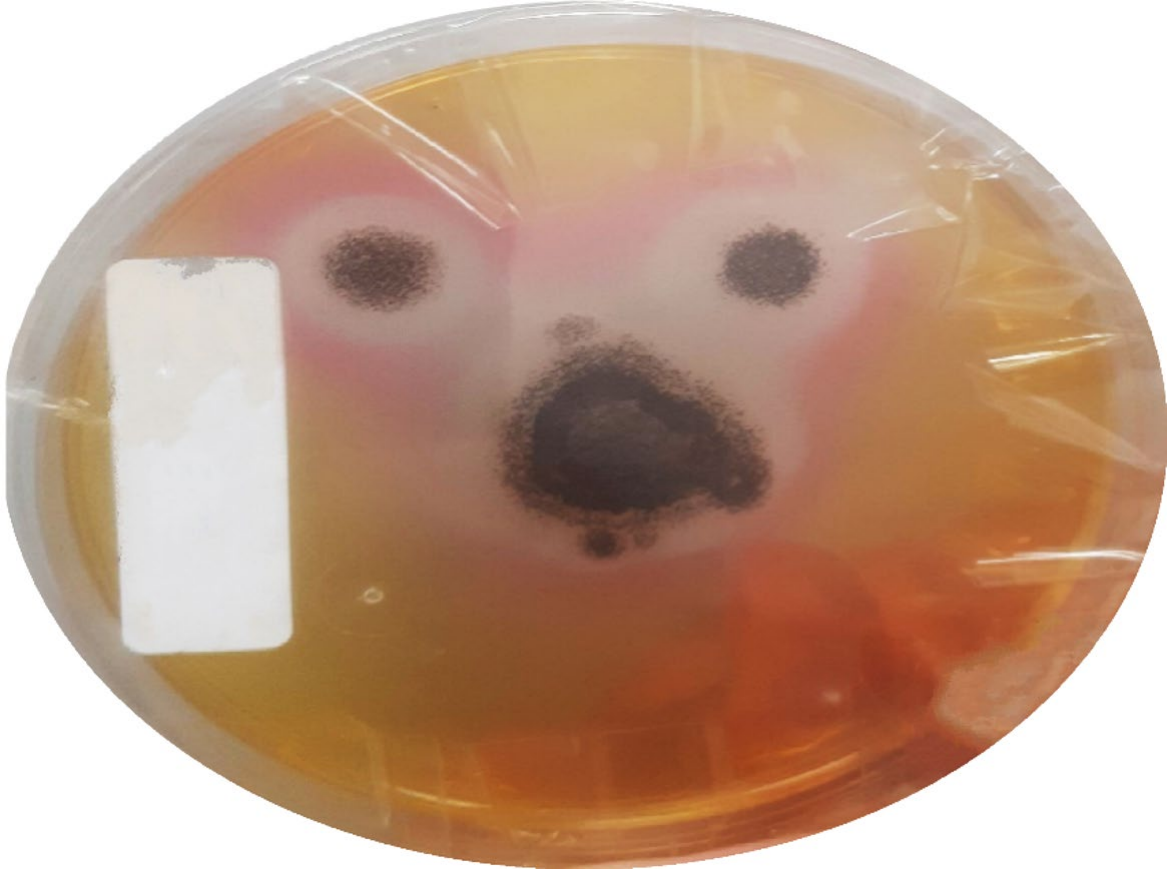
The color of the culture medium changed from orange to reddish, indicating fatty acid release due to lipase activity.

*Aspergillus niger ATCC 16,878* exhibits lipase activity. Tween 20 containing agar plates supplemented with methyl red as a pH indicator initially displayed yellow to orange coloration (pH 7). Within 48 h of fungal inoculation, a distinct reddish color developed around the colonies (Fig. 2), indicating acidification of the surrounding medium.

**Fig. 3:**
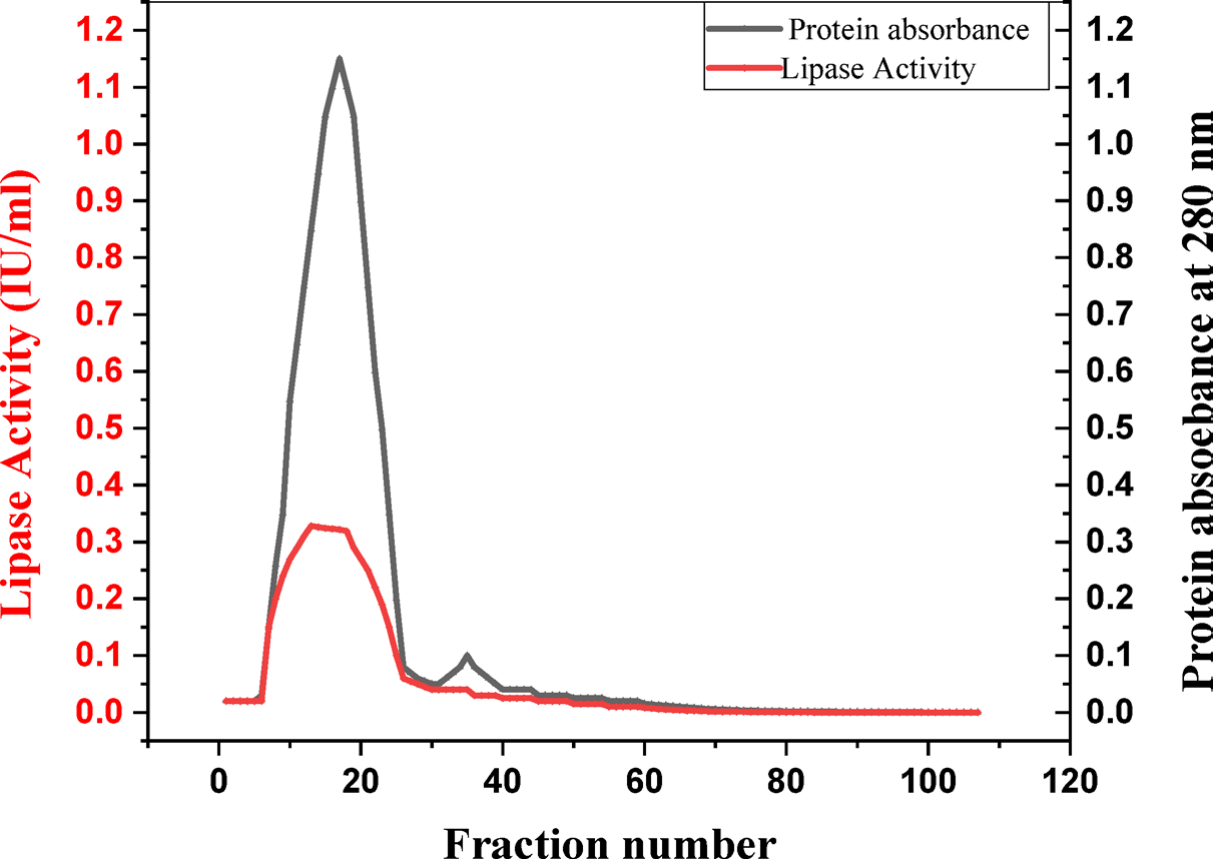
Elution profile of A. niger Lipase (gel filtration chromatography on Sephadex G-100).

Following confirmation of lipase activity, the enzyme from the *A. niger* filtrate was purified via ammonium sulfate precipitation, followed by dialysis and Sephadex G-100 size-exclusion chromatography to prepare for immobilization onto the biogenic IONPs. No visible pellet formation was observed at ammonium sulfate concentrations below 80% saturation^32,33^. The 80% fraction, which presented the highest lipase activity, was dialyzed to remove excess salt prior to further purification. The elution profile of the Sephadex G-100 column is shown in (Fig. 3). The fractions exhibiting the highest lipase activity (fractions 7–25) were pooled for subsequent analysis. This purification protocol resulted in a 3.6-fold increase in lipase purity and a final yield of 42.9% (Table 1).

**Table 1:** Complete purification profile of lipase from *A. niger* ATCC 16,878.

Fractions	Total activity (U)	Total protein (mg)	Specific activity (U/mg)	Yield %	Purification Fold
Fungal Crude Extract	2832	26.5	105	100	1
80% Ammonium sulphate precipitation	2213	13.3	166.3	78.1	1.58
Column chromatography (Sephadex G-100)	1216	3.2	380	42.9	3.6

(U) represents the amount of p-nitrophenol (µmol) produced per minute under standard assay.

### Biosynthesis of biogenic iron oxide nanoparticles (IONPs)

Iron oxide nanoparticles (IONPs) were synthesized via the use of a cell-free filtrate of *Aspergillus niger* ATCC 16,878, and the initial pale-yellow filtrate rapidly transitioned to a reddish-brown color upon the addition of iron (III) chloride hexahydrate FeCl_3_ ·6H_2_O and iron (II) sulfate heptahydrate FeSO_4_ ·7H_2_O, and the pH was adjusted to 9.0. In contrast, the control sample (filtrate only, no iron salts) remained clear. The reddish-brown color observed in the treatment group was consistent with the formation of iron oxide nanoparticles (Supplementary Fig. S1).

### Characterization of IONP

**Fig. 4:**
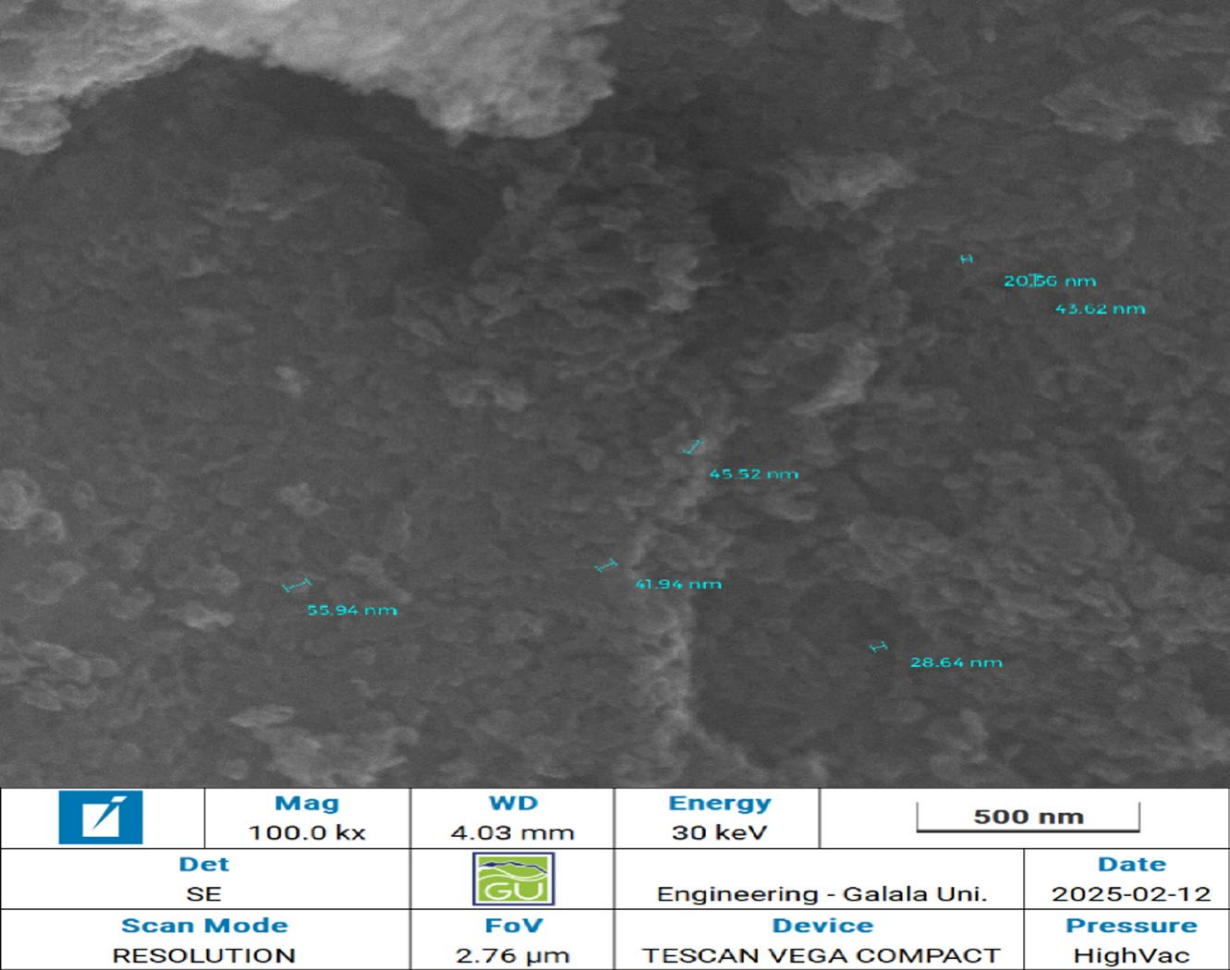
SEM image of biosynthesized IONPs at 100 KX Mag, Showing clusters as well as individual IONPs.

**Fig. 5:**
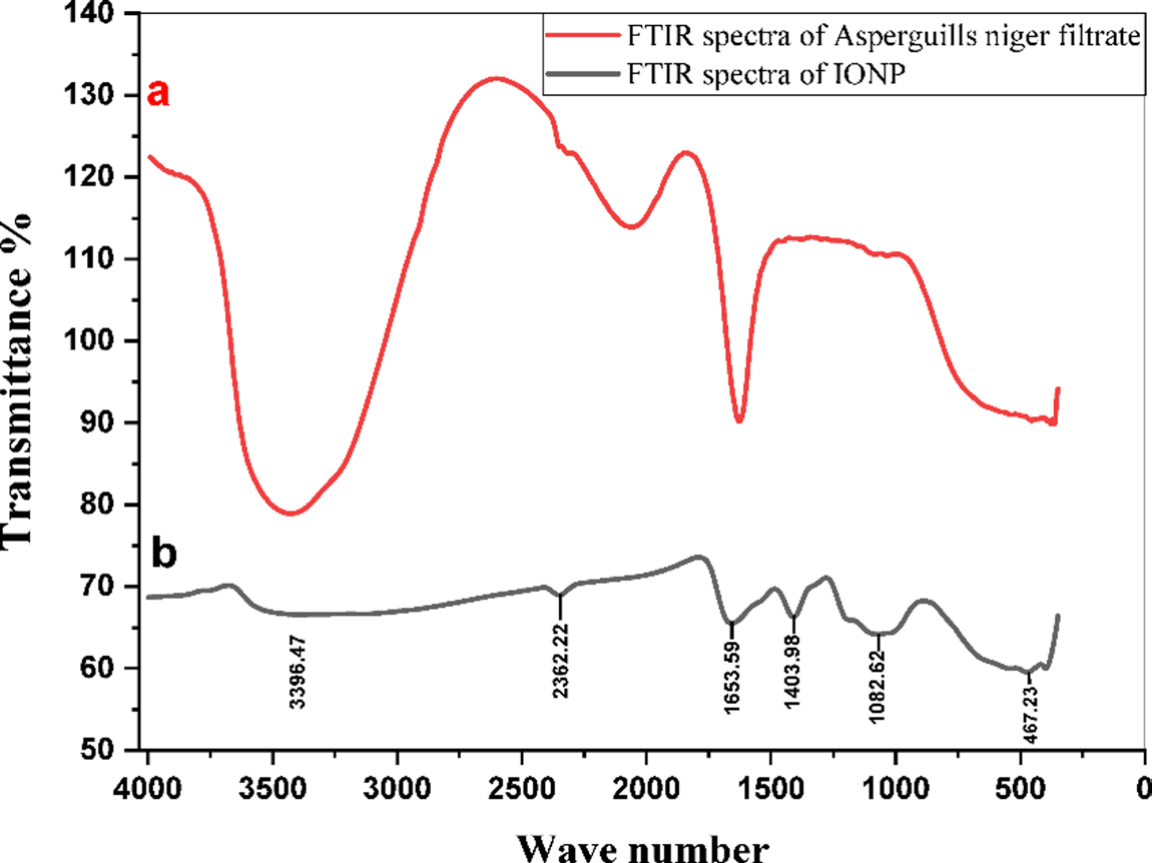
FTIR spectrum of (**a**) Asperguills niger filtrate. (**b**) Biosynthesized IONPs.

**Fig. 6:**
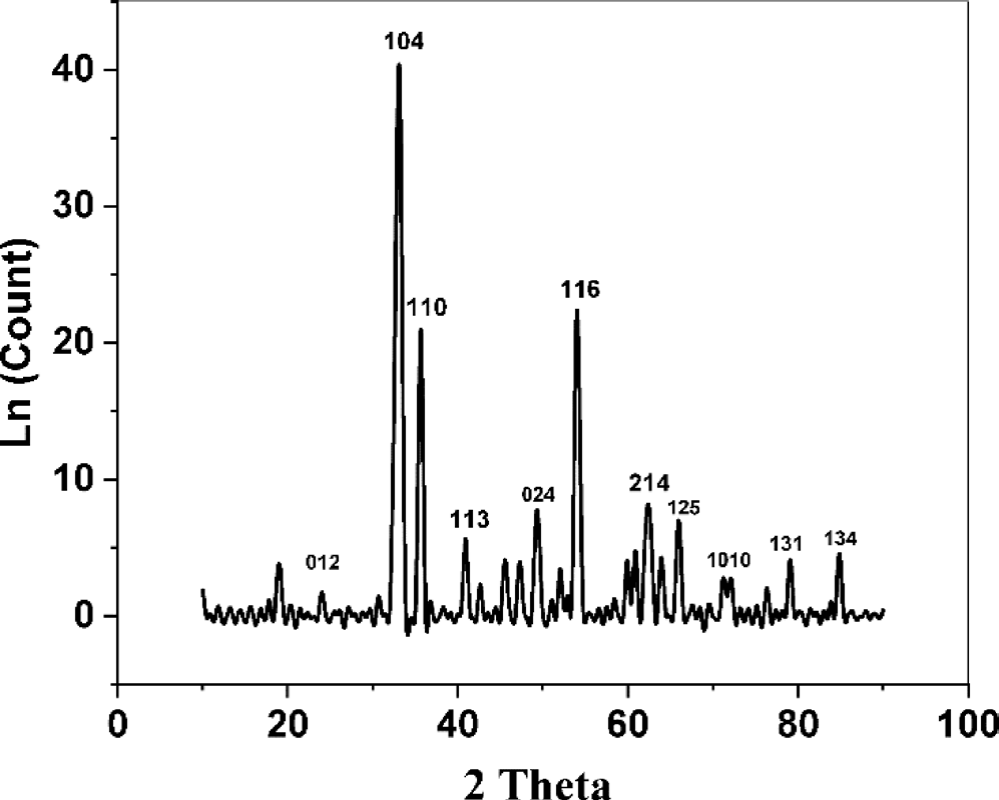
XRD spectrum of biosynthesized iron oxide nanoparticles.

The successful biosynthesis of iron oxide nanoparticles (IONPs) was initially confirmed by UV-Vis spectroscopy, displaying the characteristic absorption peak at λmax 310 nm (Supplementary Fig. S2). Nanoparticle morphology was characterized in detail using SEM. At high magnification, the IONPs were observed as individual nanoparticles predominantly quasi-spherical morphology, ranging in size from 20 to 55 nm (Fig. 4). At lower magnifications, these individual nanoparticles were observed to form larger, micro-scale aggregates (Supplementary Fig. S.3). Further characterization by FTIR (Fig. 5), revealed C-O, C = C, N-H, and Fe-O vibrations, indicating surface-capping biomolecules, and the results were also cross-referenced with those extracted from fungi. XRD (Fig. 6), (JCPDS 33–664, 18.3 nm crystallite size) revealed a crystalline structure. EDX (Supplementary Fig. S4) revealed high iron (44.40 wt%) and oxygen (27.02 wt%) contents, and the data and results of the composition were used to better correlate the carbon values with the presence of biomolecules. VSM (Supplementary Fig. S5) revealed superparamagnetic to ferromagnetic behavior, a property that is maintained at the core and reused for catalysis to separate without material loss for industrial testing.

### Lipase immobilization

**Fig. 7:**
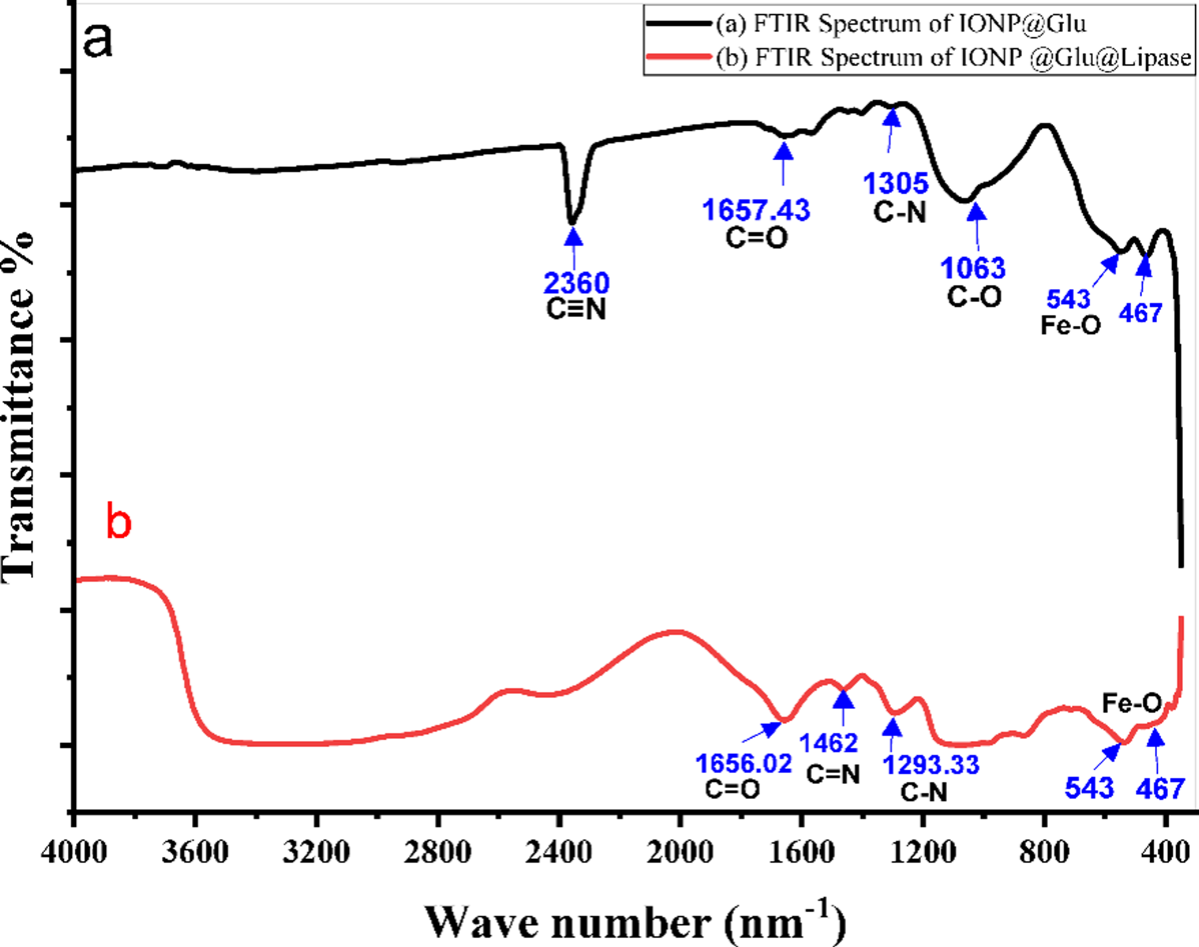
FTIR spectrum of (**a**) IONP @ Glutaraldehyde. (**b**) IONP @ Glutaraldehyde @ Lipase.

Purified lipase was immobilized onto *A. niger*-derived IONPs using glutaraldehyde as the crosslinking agent (Supplementary Fig. S6). The immobilization process achieved 81.73% efficiency and 97.4% recovery activity (Table 2). FTIR spectroscopy verified the surface modification of IONPs following glutaraldehyde activation and lipase immobilization. Shifts in the functional group vibrations confirmed successful covalent bonding and Schiff base formation between the IONPs and immobilized lipase (Fig. 7).

**Table 2:** Immobilization parameters.

Parameter	Immobilization Yield (IY)	Enzyme offered per gram of support (Atoff)	Theoretical Activity (AtT)	Immobilized Enzyme Activity (AtD)	Recovery Activity (AtR)
Value	81.73%	13,140 U/g support	10,732 U/g support	10,500 U/g support	97.4%

### Enzyme kinetics and effect of pH, temperature, reusability

**Fig. 8:**
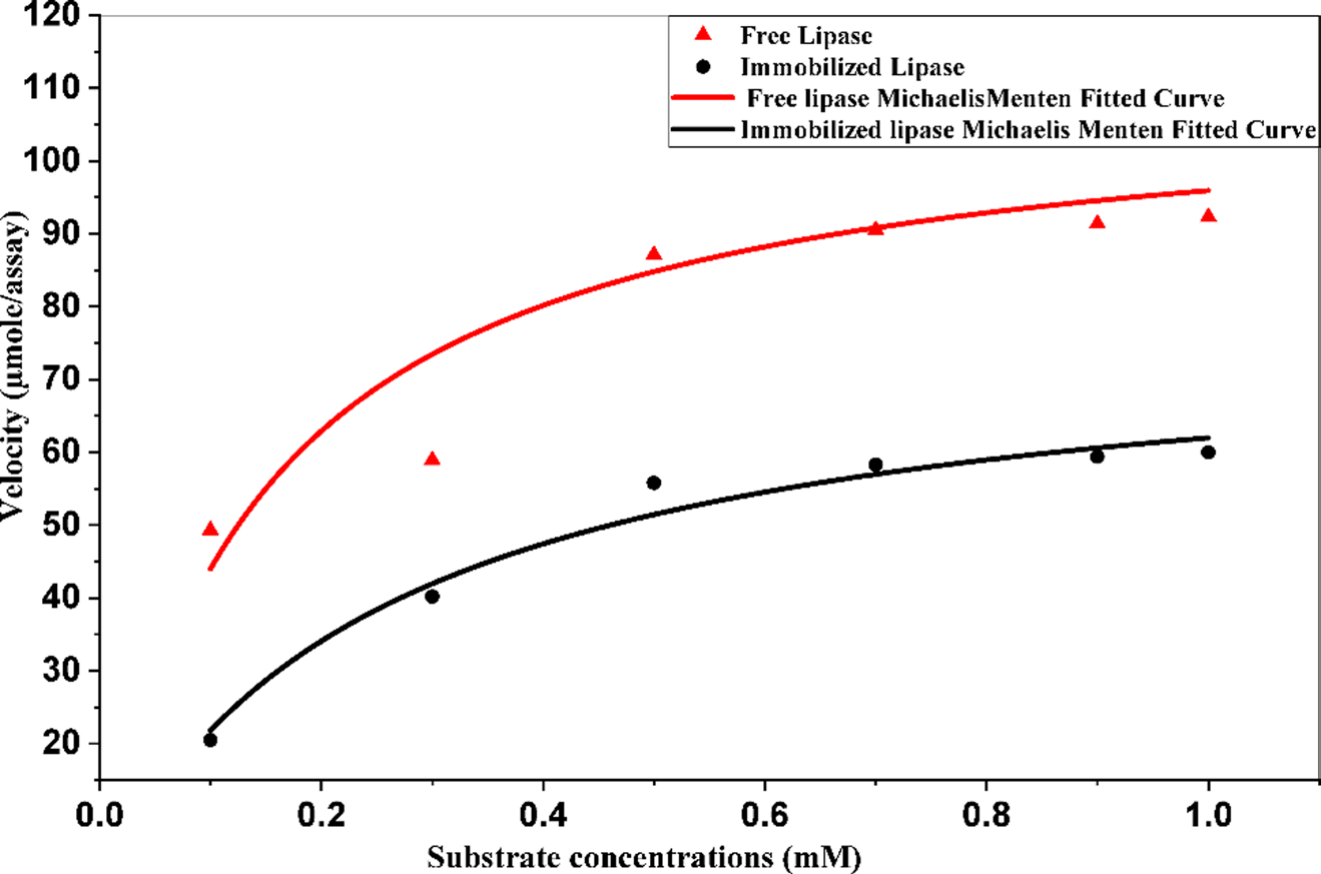
Michaelis‒Menten nonlinear fitting plots for the KM and max values of *Aspergillus niger* ATCC 16,878 lipase in the presence of various concentrations of pNPP.

Kinetic analysis revealed the expected trade-offs and benefits of immobilization (Fig. 8). The immobilized lipase showed an increased Km (from 0.147 to 0.287 mM), while the Vmax decreased from 106.29 µmol/assay (free enzyme) to 80.56 µmol/assay (immobilized enzyme), confirming that absolute activity is reduced upon immobilization.

**Fig. 9:**
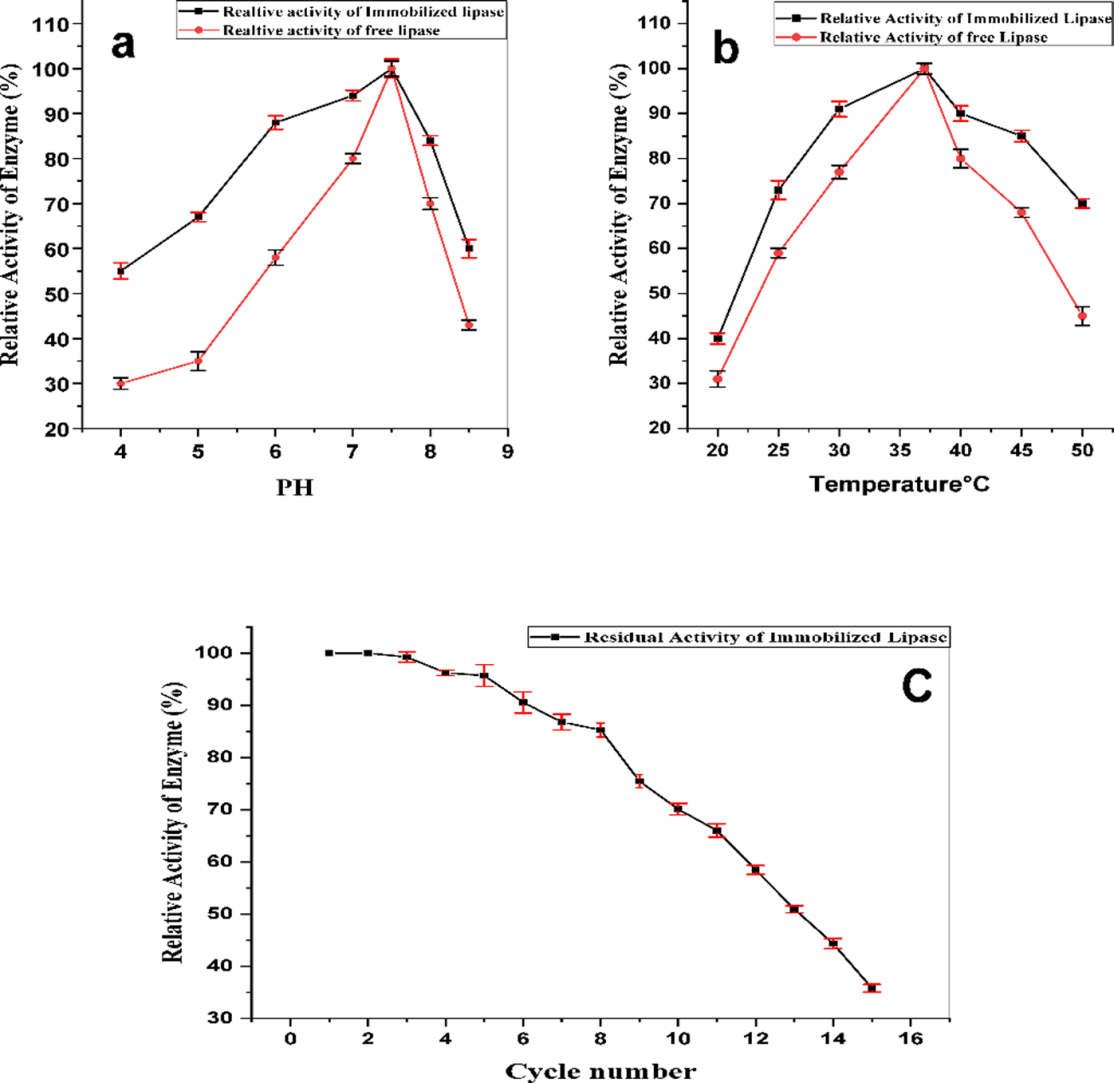
Catalytic activity of free and immobilized lipases. (**a**) As a function of pH, (**b**) as a function of temperature, and (**c**) as a function of reusability for the immobilized lipase, the relative activity of each enzyme form is normalized to its own maximum activity (100%).

However, despite this decrease in maximum velocity, the nanobiocatalyst demonstrated significantly enhanced operational stability across wider pH and temperature ranges (Fig. 9a, b). For example, at pH 8, the immobilized lipase retained approximately 80% of its optimal activity, whereas the free enzyme retained only 65% of its. Similarly, the immobilized lipase showed greater thermal stability; at 40 °C, it retained ~ 90% of its maximal activity, compared to only ~ 80% for the free enzyme. This improved stability at suboptimal conditions is a key advantage of the immobilized system.

The reusability of the nanobiocatalyst was evaluated over 15 consecutive cycles (Fig. 9c). The immobilized lipase maintained over 80% of its initial activity after eight cycles and retained approximately 35% of its activity by the 15th cycle, demonstrating significant.

operational stability.

### Effect of different storage temperatures

The free lipase was stable at 4 °C after 18 days of incubation; it retained 94.76% of the relative activity and reached a minimal activity of 30% after 90 days. After 18 days of incubation at 25 °C, 60.2% of the relative activity was retained, reaching 33% after 60 days. The immobilized lipase showed greater stability at 4 °C after the same 18 days; it retained 97% of the relative activity, while it reached 63% of the relative activity after 90 days. At 25 °C, the relative activity after 18 days was 66.8%. The percentage subsequently reached 6% after 80 days. As shown in Supplementary Fig. S7.

### Dye degradation assays (Methyl orange and methylene blue)

**Fig. 10:**
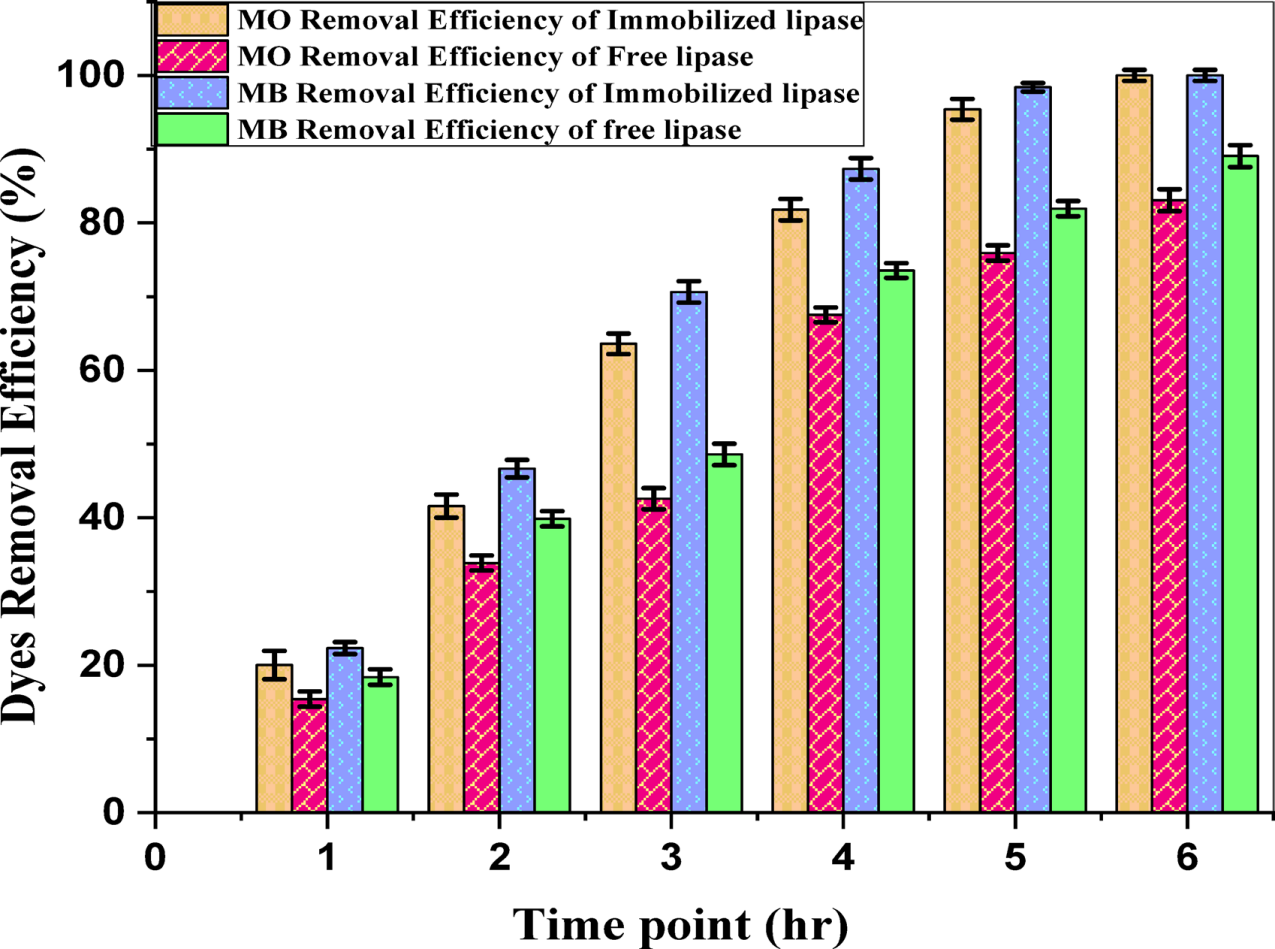
Methyl Orange (MO) and Methylene Blue (MB) degradation in terms of Removal Efficiency (%) over time, Data represent mean ± SD (*n* = 3).

The immobilized and free *A. niger* lipase degraded both methyl orange (MO) and methylene blue (MB) over 6 h, with the immobilized form consistently outperforming the free lipase (Fig. 10). At 1 h, the immobilized lipase achieved 20.03% (MO) and 22.33% (MB) removal versus 15.4% and 18.4%, respectively, for free lipase. After 4 h, the immobilized lipase achieved 81.8% (MO) and 87.33% (MB) removal, compared with 67.53% and 73.6%, respectively, for free lipase. The immobilized lipase achieved nearly complete removal at 6 h: 95.41% (MO) and 98.5% (MB). The decolorization rates were initially higher for the immobilized lipase, peaking at 2–3 h for MO and 3–4 h for MB (Supplementary Fig. S8).

### Evaluation of oil stain removal from fabric

The efficacy of oil-stain removal via free and immobilized lipases, with or without detergent supplementation, was visually assessed across the seven experimental groups. Table 3 summarizes the qualitative observations of fabric destaining posttreatment, ranked by increasing effectiveness from Group 1 (untreated control) to Group 7 (detergent + immobilized lipase).

**Table 3:** Visual assessment of oil stain removal across experimental groups.

GP	Treatment	Deep frying oil	Visual observation
1	Control (Dryed oil stain)		Fabric retained the full oil stain with no visible reduction in intensity.
2	Control (Water wash (1 h)		Minimal destaining observed; fabric retained a saturated oily residue.
3	Water + Detergent		Partial removal of oil, with lighter residual staining compared to Group 2.
4	Water + Free Lipase		Moderate destaining; faint discoloration persisted, indicating incomplete hydrolysis.
5	Water + Immobilized Lipase		Significant stain reduction; only trace residues visible.
6	Water + Detergent + Free lipase		Near-complete destaining with minor residual marks.
7	Water + Detergent + Immobilized Lipase		Nearly stain-free fabric, demonstrating optimal oil degradation.

## Discussion

The production and purification of lipase from *Aspergillus niger* ATCC 16,878 provides the foundation for our single-species nanobiocatalytic strategy. Initial screening using Tween 20 agar plates with methyl red efficiently demonstrated the lipolytic potential of the fungal strain through a rapid color change attributed to the enzymatic hydrolysis of Tween 20 and subsequent fatty acid release. This visual confirmatory technique is a reliable method for identifying lipase-producing microorganisms^36^.

Our two-step purification strategy involving ammonium sulfate precipitation followed by Sephadex G-100 size-exclusion chromatography demonstrated remarkable efficacy in isolating lipases. The selection of 80% ammonium sulfate saturation was crucial, facilitating selective protein precipitation and effectively differentiating the target lipase from other cellular proteins^32,33^. Chromatographic separation via Sephadex G-100 further refined the purification process, with peak lipase activity observed in fractions 7–25.

The purification protocol achieved 3.6-fold purification with a 42.9% protein yield, this result matches with similar lipase purification studies from Aspergillus species^22,35^. This outcome not only validates our methodology but also underscores the potential of *A. niger* ATCC 16,878 as a robust lipase source. The combination of ammonium sulfate precipitation and size-exclusion chromatography has emerged as a powerful approach for obtaining purified enzyme preparations that are suitable for subsequent investigations.

The ability to efficiently produce and purify lipases enables the integration of multiple biotechnological applications from the same microbial source. To achieve successful lipase production, we utilized *A. niger* filtrate for the eco-friendly synthesis of iron oxide nanoparticles (IONPs). The rapid color transition from pale yellow to reddish-brown upon mixing the fungal filtrate with iron salts provided initial confirmation of IONP formation^36,37^. This color change, was not observed in the control experiment, directly indicated the reduction of iron precursors by fungal filtrate components^7,38,39^. UV‒Vis spectroscopy confirmed this finding, with a peak at 310 nm, which is consistent with that of green-synthesized IONPs^40–44^. Our approach leverages the eco-friendly and cost-effective advantages of biogenic synthesis while maintaining a single-species strategy that is central to this study.

FTIR analysis of the biosynthesized IONPs confirmed the presence of fungal biomolecules, as indicated by shared peaks with the *A. niger* filtrate at the C-O, C = C, and N-H stretches (1082.62, 1403.98, and 1653.59 cm⁻¹, respectively). These findings suggest a role for fungal proteins, polysaccharides, or aromatics in nanoparticle capping and stabilization 43). Characteristic Fe‒O vibrations (534 and 476 cm⁻¹) confirmed iron oxide formation, with data consistent with previous work, whereas the absence of precursor peaks (ferrous sulfate ~ 1000 cm⁻¹, ferric chloride ~ 739 cm⁻¹) further supported successful IONP synthesis^35,37^.

XRD analysis revealed that the IONPs were hematite (α-Fe2O3), with a clear peak at 24.14^0^, with all others indexed to the (012), (104), (110), (024), and (116) planes (JCPDS card numbers (33–664), which is in agreement with the findings of others who were able to achieve synthesis^7,46^. The average crystallite size (18.3 nm) was calculated by the Debye–Scherrer formula via the peak at 33.4^0^, corresponding to (104), which is within the value expected by others for such nanoparticles^44–47^.

The morphology of the biosynthesized IONPs was confirmed by SEM imaging, which showed primary particles with a quasi-spherical shape, consistent with other mycosynthesized IONPs^49,50^. While the particles showed a tendency to form aggregates, a common characteristic of magnetic nanoparticles due to their intrinsic dipole-dipole interactions, the presence of capping biomolecules from the fungal filtrate likely plays a crucial role in mitigating more extensive agglomeration. It is a known advantage of green synthesis that these biological capping agents can enhance colloidal stability compared to nanoparticles produced by some conventional chemical routes^43^. This is further supported by the EDX confirmed high iron (44.40 wt%) and oxygen (27.02 wt%) contents, which are indicative of iron oxide^51^, where the presence of carbon (21.12 wt%) indicates the incorporation of biomolecules from the *A. niger* filtrate^13,52,53^.

VSM analysis revealed that the data obtained from the sample exhibited superparamagnetic to ferromagnetic behavior, with a saturation magnetization (Ms) of 2.4944 emu/g. This value is notably lower than that of bulk magnetite (~ 92 emu/g)^54^, possibly because of surface spin disorder, reduced crystallinity, or the presence of organic material^48,55^.

It is important to note that the size of these nanoparticles is a critical parameter for enzyme immobilization, as it directly influences the surface area available for enzyme loading. While not optimized in this initial study, our biogenic synthesis method offers clear pathways for tuning the particle diameter. The final size is governed by the ratio between the iron salt precursors and the concentration of biomolecules in the fungal filtrate. A higher concentration of filtrate would likely favor the nucleation of many small crystals, leading to smaller nanoparticles, whereas a higher concentration of iron salts would favor crystal growth, resulting in larger particles. This principle of size-controllability is a known advantage of fungal-mediated synthesis^38^ and represents a key avenue for future optimization of this nanobiocatalytic system.

The success of the subsequent immobilization step is a critical validation of our single-source strategy. In conventional multi-source systems, as highlighted in Supplementary Table S1, enzymes are often immobilized on foreign supports, which can lead to suboptimal activity retention. For instance, Osuna et al.^56^ immobilized *A. niger* lipase onto chemically synthesized nanoparticles and retained only 64.4% of the enzyme’s activity. Even when using advanced, state-of-the-art supports, the limitations of a multi-source approach are evident. Xia et al.^57^ used a sophisticated BioMOF support for the same *A. niger* lipase and achieved an activity recovery of 89.2%, while Li et al.^58^ reached ~ 95% with a graphene-based system. In stark contrast, our simple, biogenic, single-source system retained a remarkable 97.4% of its activity and immobilization yield of 81.73%, surpassing all of these conventional approaches. We hypothesize that this superior functional preservation is due to the high biochemical affinity between the lipase and the biomolecules capping the IONPs, both produced from the same *A. niger* species. This inherent compatibility appears to be a more critical factor for preserving enzyme function than the complexity of the support material itself. This conclusion is further supported by FTIR analysis of the immobilized system confirmed successful surface modification and enzyme attachment. The spectral changes, including shifts in the C = O stretch and the appearance of new C‒N bands, provided strong evidence for covalent bonding between lipase and glutaraldehyde-modified IONPs^57,58^.

The exceptional compatibility between the enzyme and the support material, both derived from the same fungal source, appears to be a key factor in the successful development of this nanobiocatalyst system.

The immobilization of *A. niger* lipase onto biogenic IONPs resulted in notable changes in enzyme kinetics, with an increase in km (0.150 to 0.343 mM) and a decrease in Vmax (110.4 to 88.8 µmol/assay). These modifications, which are typical of immobilized systems, reflect structural adaptations at the enzyme-support interface^35^. However, the immobilized enzyme demonstrated enhanced operational characteristics that offset these kinetic alterations.

A key advantage of our nanobiocatalyst system is its expanded pH stability profile. While free lipase showed optimal activity at pH 7.5, the immobilized form maintained approximately 80% relative activity at pH 8.5, demonstrating superior alkaline tolerance^59–61^. This enhanced pH stability, surpassing previous reports^62–64^, suggests effective protection of the enzyme structure by the IONP support. Similarly, both forms exhibited optimal activity at 37 °C, with the immobilized enzyme showing improved stability owing to the protective microenvironment created by the nanoparticle support^65^.

Another key advantage of our nanobiocatalyst is its operational stability, as demonstrated by its performance over repeated cycles. The immobilized lipase retained over 80% of its initial activity after eight consecutive uses, a result that compares favorably with or surpasses previously reported results for various immobilized lipases, including *Thermomyces lanuginosus*^66^, *Serratia marcescens*^67^, and *Candida antarctica*^68^. The gradual decline in activity to approximately 35% by the 15th cycle is a common phenomenon for immobilized enzymes, even when covalently bound. While the strong covalent linkage minimizes enzyme leaching, this progressive loss of function is more likely attributable to other factors. These include subtle conformational changes or partial denaturation of the enzyme’s active site from repeated exposure to the substrate and reaction conditions, mechanical stress during the magnetic recovery and washing steps, or potential fouling of the active site. Nevertheless, the ability to maintain high activity for at least eight cycles represents a significant improvement over the single-use capability of the free enzyme and confirms the practical viability of our single-source nanobiocatalyst.

The storage stability of free and immobilized lipases was investigated. After 18 days at storage temperatures of 4 °C and 25 °C, the immobilized lipase retained 97% and 66% of its relative activity, respectively. The free lipase content decreased to 89% and 56%, respectively, indicating that the constructed covalent bonds created a stable environment for the lipase^69^.

The widespread presence of synthetic dyes such as methyl orange (MO), methylene blue (MB), and other lipid-based products in industrial wastewater necessitates efficient and sustainable remediation strategies^70^. Although conventional methods often have high energy requirements and produce toxic byproducts^71^, nano biocatalytic systems offer promising alternatives. This study demonstrated the superior efficacy of an *A. niger*-derived, single-source nano biocatalyst for both dye degradation and oil stain removal, with data collected indicating that an efficient system that has greater than or equal performance when tested over free lipase and control groups.

The immobilized lipase achieved near-quantitative removal of both MO (95.41%) and MB (98.5%) within 6 h, exceeding the performance of free lipase. This result also compares favorably with previous studies using conventional multi-source systems. For example, Yao et al.^72^ used a lipase immobilized on chemically synthesized graphene oxide and reported a maximum removal of 89.47% for Methyl Orange after 4 h. Our system achieved a complete degradation of MO and demonstrated high efficiency against Methylene Blue. This enhanced degradative performance is consistent with the high activity retention and operational stability conferred by our novel single-source synthesis method, highlighting the practical benefits of the catalyst developed in this work.

The faster initial kinetics of the immobilized system (20–22% removal at 1 h vs. 15–18% for free lipase) likely reflect the enhanced enzyme stability and substrate binding facilitated by IONP immobilization^73^. MO degradation likely proceeds via azoreductase-mediated cleavage of the azo bond^74^, whereas MB degradation (also 98.5%) suggests oxidative breakdown of the thiazine ring, potentially via laccase-like activity associated with fungal lipases^75^. The contrasting peak discoloration rates (MB: 3–4 h; MO: 2–3 h) highlight the influence of the dye structure.

Furthermore, visual analysis of oil stain removal from cotton fabric demonstrated the practical utility of immobilized lipase, particularly in combination with a 1% detergent solution. The combination achieved near-complete destaining, outperforming both the free enzyme, detergent alone, and control groups, underscoring its use on the basis of the hydrolytic mechanism of the lipase enzyme^76^. Collectively, these findings highlight the potential of our single-source biocatalyst for sustainable wastewater treatment. By showing strong activity that removes pollutants without toxic byproducts, this system overcomes the challenges associated with current approaches and offers a new system that meets the requirements of a clean approach to remediate industrial waste.

## Conclusion

This study introduces a single source nano biocatalysis approach using *Aspergillus niger* ATCC 16,878, which produces both lipase and iron oxide nanoparticles (IONPs), creating a highly compatible and efficient nanobiocatalytic system. The key achievements included an 81.73% immobilization yield and 97.4% activity retention, along with enhanced operational stability, broader pH tolerance, and distinctive reusability over eight cycles. Importantly, the biocatalyst demonstrated significant potential in practical applications, effectively degrading industrial dyes in wastewater and achieving near-complete oil-stain removal from fabric. The single-source method offers streamlined production, improved enzyme-support compatibility, and environmental sustainability. These findings validate the importance of biological source compatibility and pave the way for more efficient nanobiocatalytic systems. Future work should focus on translating this strategy towards industrial application. This will involve adapting the process for the direct immobilization of crude or partially purified enzyme extracts to enhance economic feasibility, optimizing enzyme loading parameters, and scaling up the synthesis for use in continuous reactor systems. Furthermore, clarifying the dye degradation pathways and testing performance in mixed-pollutant systems will be crucial for real-world implementation.

## Supplementary Information

Below is the link to the electronic supplementary material.

Supplementary Material 1

## Data Availability

The datasets utilized and analyzed in this study are accessible from the corresponding author upon reasonable request.

## References

[1] R. Fopase, S. Sharma, and L. M. Pandey, “Nano (Bio) Catalysts: An Effective Tool to Utilize Waste Cooking Oil for the Biodiesel Production,” in Nano- and Biocatalysts for Biodiesel Production, A. P. Ingle, Ed. Chichester, UK: John Wiley & Sons, Ltd, 2021, pp. 31–51.

[2] Khafaga, D. S. R., Radwan, M. G., Muteeb, G., Aatif, M. & Farhan, M. Green synthesis of biocatalysts based on nanocarriers promises an effective role in pharmaceutical and biomedical fields. *Catalysts* **13** (11,1448). 10.3390/catal13111448 (2023).

[3] Chandra, Enespa, R., Singh, K. & Arora Microbial lipases and their industrial applications: a comprehensive review. *Microb. Cell. Factories*. **19** (1). 10.1186/s12934-020-01428-8 (2020). 10.1186/s12934-020-01428-8 PMC7449042 32847584

[4] Fernandez-Lafuente, R. Lipase from thermomyces lanuginosus: uses and prospects as an industrial biocatalyst. *J. Mol. Catal. B Enzym*. **62** (3-4), 197–212. 10.1016/j.molcatb.2009.11.010 (2010).

[5] Sari, A. N. M. & Prasetyo, E. N. M.Koentjoro, and Lipase immobilization based on biopolymer.

[6] Rodrigues, R. C. et al. Immobilization of lipases on hydrophobic supports: immobilization mechanism, advantages, problems, and solutions. *Biotechnol. Adv.* **37**, 5,746–770. 10.1016/j.biotechadv.2019.04.003 (2019). 30974154 10.1016/j.biotechadv.2019.04.003

[7] Saied, E. et al. Mycosynthesis of hematite (α-Fe2O3) nanoparticles using Aspergillus Niger and their antimicrobial and photocatalytic activities. *Bioengineering* **9** (8,397). 10.3390/bioengineering9080397 (2022). 10.3390/bioengineering9080397 PMC9404788 36004922

[8] Maghraby, Y. R., El-Shabasy, R. M., Ibrahim, A. H. & Azzazy, H. M. E. S. Enzyme immobilization technologies and industrial applications. *ACS Omega*. **8** (6), 5184–5196. 10.1021/acsomega.2c07560 (2023). 36816672 10.1021/acsomega.2c07560 PMC9933091

[9] El-Khawaga, A. M., Zidan, A. & El-Mageed, A. I. A. A. Preparation methods of different nanomaterials for various potential applications: A review. *J. Mol. Struct.* **1281** (135148). 10.1016/j.molstruc.2023.135148 (2023).

[10] Thakkar, K. N., Mhatre, S. S. & Parikh, R. Y. Biological synthesis of metallic nanoparticles. *Nanomed. Nanotechnol Biol. Med.* **6**, 2,257–262. 10.1016/j.nano.2009.07.002 (2010). 10.1016/j.nano.2009.07.002 19616126

[11] Boroumand Moghaddam, A. et al. Nanoparticles biosynthesized by fungi and yeast: a review of their preparation, properties, and medical applications, *Molecules*, 20,9, Art.9, (2015). 10.3390/molecules200916540 10.3390/molecules200916540 PMC6332129 26378513

[12] Šebesta, M. et al. Mycosynthesis of metal-containing nanoparticles—synthesis by ascomycetes and basidiomycetes and their application, *Int. J. Mol. Sci.*, 24,1, Art.1, (2023). 10.3390/ijms24010304 10.3390/ijms24010304 PMC9820721 36613746

[13] X. Z. Xiaohai Yang, Different Active Biomolecules Involved in Biosynthesis of Gold Na… Ingenta Connect. 2024. [Online]. Available: https://www.ingentaconnect.com/content/asp/jbn/2011/00000007/00000002/art00003;jsessionid=teo4l3a090bb.x-ic-live-01.

[14] Li, C., Jiang, S., Zhao, X. & Liang, H. Co-immobilization of enzymes and magnetic nanoparticles by metal-nucleotide hydrogelnanofibers for improving stability and recycling. *Molecules* **22** (1). 10.3390/molecules22010179 (2017). 10.3390/molecules22010179 PMC6155653 28125003

[15] Liang, H. et al. Co-immobilization of multiple enzymes by metal coordinated nucleotide hydrogel nanofibers: improved stability and an enzyme cascade for glucose detection. *Nanoscale* **8** (11), 6071–6078. 10.1039/C5NR08734A (2016). 26932320 10.1039/c5nr08734a

[16] Wang,Dou,Zhao, X., Zhao, C., Ding, Y. & andXu Immobilization of lipases onto magnetic fe3o4 nanoparticles for application in biodiesel production. *ChemSusChem* **2** (10), 947–950. 10.1002/cssc.200900174 (2009). 19780103 10.1002/cssc.200900174

[17] Shah, S., Solanki, K. & Gupta, M. N. Enhancement of lipase activity in non-aqueous media upon immobilization on multi-walled carbon nanotubes. *Chem. Cent. J.* **1** (1). 10.1186/1752-153X-1-30 (2007). 10.1186/1752-153X-1-30 PMC2211749 18047656

[18] Chen, M., Zeng,Xu, G., Lai, C. & Tang, L. How do enzymes ‘meet’ nanoparticles and nanomaterials? *Trends Biochem. Sci.* **42**, 11,914–930. 10.1016/j.tibs.2017.08.008 (2017). 28917970 10.1016/j.tibs.2017.08.008

[19] Samad, M. Y. A. et al. A plate assay for primary screening of lipase activity. *J. Microbiol. Methods*. **9** (1,51–56). 10.1016/0167-7012(89)90030-4 (1989).

[20] Rai, B., Shrestha, A., Sharma, S. & Joshi, J. Screening, optimization and process scale up for pilot scale production of lipase by Aspergillus Niger. *Biomed. Biotechnol.* **2**, 54–59. 10.12691/bb-2-3-3 (10 2014).

[21] Gupta,Rathi, N. & Gupta, R. Simplified para-nitrophenyl palmitate assay for lipases and esterases. *Anal. Biochem.* **311** (1), 98–99. 10.1016/S0003-2697(02)00379-2 (2002). 12441161 10.1016/s0003-2697(02)00379-2

[22] Souza, L. T. A. et al. Lipolytic potential of aspergillus japonicus LAB01: production, partial purification, and characterisation of an extracellular lipase, *BioMed Res. Int.*, 108913, 2014, (2014). 10.1155/2014/108913 10.1155/2014/108913 PMC4230215 25530954

[23] OliverH, Lowry, N. J., Rosebrough, A. L., Farr & Randall, R. J. Protein measurement with the Folin phenol reagent. *J. Biol. Chem.* **193,1**, 265–275. 10.1016/S0021-9258(19)52451-6 (1951). 14907713

[24] Adham, N. Z. & Ahmed, E. M. Extracellular lipase of Aspergillus Niger NRRL3; production, partial purification and properties. *Indian J. Microbiol.* **49**, 1,77–83. 10.1007/s12088-009-0004-2 (2009). 23100754 10.1007/s12088-009-0004-2 PMC3450051

[25] Sharma, N., Sharma, S., Pathania & Handa, S. Purification and characterization of lipase by Bacillus Methylotrophicus PS3 under submerged fermentation and its application in detergent industry. *J. Genet. Eng. Biotechnol.* **15** (2), 369–377. 10.1016/j.jgeb.2017.06.007 (2017). 30647675 10.1016/j.jgeb.2017.06.007 PMC6296573

[26] Balan, A., Ibrahim, D., Rahim, R. A. & Ahmad Rashid, F. A. Purification and characterization of a thermostable lipase from *Geobacillus thermodenitrificans* IBRL-nra. *Enzyme Res.* **2012**, 1–7. 10.1155/2012/987523 (2012). 10.1155/2012/987523 PMC3503269 23198138

[27] Layne, E. C. Spectrophotometric and turbidimetric methods for measuring proteins. *Methods Enzymol.* **3**, 447–454 (1957).

[28] Abosharaf, H., Radwan, M. G., Abou-Saleh, R. H. & Mohamed, T. M. Green fabrication of iron oxide nanoparticles utilizing Aspergillus Niger. *Delta J. Sci.* **49** (2), 69–81. 10.21608/djs.2024.339304.1202 (2024).

[29] Silva, J. A., Macedo, G., Rodrigues, D. S., Giordano, R. L. C. & Gonçalves, L. R. B. Immobilization of Candida Antarctica lipase B by covalent attachment on chitosan-based hydrogels using different support activation strategies. *Biochem. Eng. J.* **60**, 16–24. 10.1016/j.bej.2011.09.011 (2012).

[30] Page, A. A non-linear regression program in BASIC for estimating Km and vmax. *Bioinformatics* **3** (1), 49–51. 10.1093/bioinformatics/3.1.49 (1987). 10.1093/bioinformatics/3.1.49 3453214

[31] Narwal, S. K., Saun, N. K. & Gupta, R. Characterization and catalytic properties of free and silica-bound lipase: a comparative study. *J. Oleo Sci.* **63** (6), 599–605. 10.5650/jos.ess13231 (2014). 24829134 10.5650/jos.ess13231

[32] Sri Kaja, B., Lumor, S., Besong, S., Taylor, B. & Ozbay, G. Investigating enzyme activity of immobilized candida rugosa lipase, *J. Food Qual.*, 1–9, 2018, (2018). 10.1155/2018/1618085

[33] Kalantari, M., Kazemeini, M., Tabandeh, F. & Arpanaei, A. Lipase immobilisation on magnetic silica nanocomposite particles: effects of the silica structure on properties of the immobilised enzyme. *J. Mater. Chem.* **22** (17,8385). 10.1039/c2jm30513e (2012).

[34] Mhetras, N. C., Bastawde, K. B. & Gokhale, D. V. Purification and characterization of acidic lipase from *Aspergillus Niger* NCIM 1207. *Bioresour Technol.* **100,3**, 1486–1490. 10.1016/j.biortech.2008.08.016 (2009). 18835775 10.1016/j.biortech.2008.08.016

[35] Ezenwelu, C. O. et al. Studies on Properties of Lipase Produced from Aspergillus sp. Isolated from Compost Soil, *Adv. Enzyme Res.*, 10,2, Art.2, (2022). 10.4236/aer.2022.102003

[36] Singh, R., Gupta, N., Goswami, V. K. & Gupta, R. A simple activity staining protocol for lipases and esterases. *Appl. Microbiol. Biotechnol.* **70**, 6,679–682. 10.1007/s00253-005-0138-z (2006). 16170531 10.1007/s00253-005-0138-z

[37] El-Ghonemy, D. H. et al. Thermo-alkali-stable lipase from a novel *Aspergillus Niger* : statistical optimization, enzyme purification, immobilization and its application in biodiesel production. *Prep Biochem. Biotechnol.* **51** (3), 225–240. 10.1080/10826068.2020.1805759 (2021). 32808876 10.1080/10826068.2020.1805759

[38] Chatterjee, S., Mahanty, S., Das,Chaudhuri & Das, S. Biofabrication of iron oxide nanoparticles using manglicolous fungus Aspergillus Niger BSC-1 and removal of Cr(VI) from aqueous solution. *Chem. Eng. J.* **385** (123790). 10.1016/j.cej.2019.123790 (2020).

[39] Sidkey, N. Biosynthesis, characterization and antimicrobial activity of iron oxide nanoparticles synthesized by fungi. *Al-Azhar J. Pharm. Sci.* **62**, 2,164–179. 10.21608/ajps.2020.118382 (2020).

[40] Hasanin, M., Hashem, A. H., Lashin, I. & Hassan, S. A. M. In vitro improvement and rooting of banana plantlets using antifungal nanocomposite based on myco-synthesized copper oxide nanoparticles and starch. *Biomass Convers. Biorefinery*. **13** (10), 8865–8875. 10.1007/s13399-021-01784-4 (2023).

[41] Zúñiga-Miranda, J. et al. Iron oxide nanoparticles: green synthesis and their antimicrobial activity. *Nanomaterials* **13** (22), 2919. 10.3390/nano13222919 (2023). 37999273 10.3390/nano13222919 PMC10674528

[42] Bouafia, A. & Laouini, S. E. Green synthesis of iron oxide nanoparticles by aqueous leaves extract of *Mentha pulegium* L.: effect of ferric chloride concentration on the type of product. *Mater. Lett.* **265** (127364). 10.1016/j.matlet.2020.127364 (2020).

[43] Karpagavinayagam & Vedhi, C. Green synthesis of iron oxide nanoparticles using *Avicennia Marina* flower extract. *Vacuum* **160**, 286–292. 10.1016/j.vacuum.2018.11.043 (2019).

[44] Haris, M. et al. Oscillatoria limnetica mediated green synthesis of iron oxide (Fe2O3) nanoparticles and their diverse in vitro bioactivities. *Molecules* **28** (5), 2091. 10.3390/molecules28052091 (2023). 36903337 10.3390/molecules28052091 PMC10004046

[45] Mathur, N., Paliwal, A., Mathur,Sharma, N. & andBhatnagar Characterization of antimicrobial compounds from Streptomyces isolates, *J. Chem. Pharm. Res.*, 7, [Online]. (2015). Available: https://api.semanticscholar.org/CorpusID:36874805

[46] Naz, S. et al. Green synthesis of hematite (α-Fe2O3) nanoparticles using *Rhus punjabensis* extract and their biomedical prospect in pathogenic diseases and cancer. *J. Mol. Struct.* **1185**, 1–7. 10.1016/j.molstruc.2019.02.088 (2019).

[47] Zhang, W., Rittmann, B. & Chen, Y. Size effects on adsorption of hematite nanoparticles on e. coli cells. *Environ. Sci. Technol.* **45** (6), 2172–2178. 10.1021/es103376y (2011). 21341780 10.1021/es103376y

[48] Abdeen, M., Sabry, S., Ghozlan, H., El-Gendy, A. A. & Carpenter, E. E. microbial-physical synthesis of Fe and Fe3O4 magnetic nanoparticles using Aspergillus Niger YESM1 and supercritical condition of ethanol. *J. Nanomater*. **2016** (9174891). 10.1155/2016/9174891 (2016).

[49] Tarafdar, J. C. & Raliya, R. Rapid, low-cost, and ecofriendly approach for iron nanoparticle synthesis using *Aspergillus oryzae* TFR9. *J. Nanopart.* **2013**, 1–4. 10.1155/2013/141274 (2013).

[50] Aziz, W. J., Abid, M. A., Kadhim, D. A. & Mejbel, M. K. Synthesis of iron oxide (β-fe2o3) nanoparticles from Iraqi grapes extract and its biomedical application. *IOP Conf. Ser. Mater. Sci. Eng.* **881** (1,012099). 10.1088/1757-899X/881/1/012099 (2020).

[51] Laurent, S. et al. Magnetic iron oxide nanoparticles: synthesis, stabilization, vectorization, physicochemical characterizations, and biological applications. *Chem. Rev.* **108** (6), 2064–2110. 10.1021/cr068445e (2008). 18543879 10.1021/cr068445e

[52] Wu, W., Wu, Z., Yu, T., Jiang, C. & Kim, W. S. Recent progress on magnetic iron oxide nanoparticles: synthesis, surface functional strategies and biomedical applications. *Sci. Technol. Adv. Mater.* **16** (2,023501). 10.1088/1468-6996/16/2/023501 (2015). 10.1088/1468-6996/16/2/023501 PMC5036481 27877761

[53] Ahmed, A., Usman, M., Yu, B., Shen, Y. & Cong, H. Sustainable fabrication of hematite (α-Fe2O3) nanoparticles using biomolecules of Punica granatum seed extract for unconventional solar-light-driven photocatalytic remediation of organic dyes. *J. Mol. Liq*. **339** (116729). 10.1016/j.molliq.2021.116729 (2021).

[54] Ioncica, M. C., Bandyopadhyay, S., Bali, N., Socoliuc, V. & Bernad, S. I. Investigation of cubic and spherical ionps’ rheological characteristics and aggregation patterns from the perspective of magnetic targeting. *Magnetochemistry* **9** (4). 10.3390/magnetochemistry9040099 (2023).

[55] Guardia et al. Surfactant effects in magnetite nanoparticles of controlled size, *Proc. Jt. Eur. Magn. Symp.*, 316,2,e756–e759, 2007. 10.1016/j.jmmm.2007.03.085

[56] Osuna, Y. et al. Immobilization of Aspergillus Niger lipase on chitosan-coated magnetic nanoparticles using two covalent-binding methods. *Bioprocess. Biosyst Eng.* **38**, 8,1437–1445. 10.1007/s00449-015-1385-8 (2015). 25759161 10.1007/s00449-015-1385-8

[57] Xia, G. et al. Preparationofa nanobiocatalyst by efficientl y immobilizing Aspergillus Niger lipase onto magnetic metal biomolecule frameworks(BioMOF). *ChemCatChem* **9** (10), 1794–1800. 10.1002/cctc.201700070 (2017).

[58] Li, X. et al. One-pot Polylol synthesis of graphene decorated with size- and density-tunable Fe3O4 nanoparticles for Porcine pancreatic lipase immobilization. *Carbon* **60**, 488–497. 10.1016/j.carbon.2013.04.068 (2013).

[59] Freire, T. M. et al. Fast ultrasound assisted synthesis of chitosan-based magnetite nanocomposites as a modified electrode sensor. *Carbohydr. Polym.* **151**, 760–769. 10.1016/j.carbpol.2016.05.095 (2016). 27474623 10.1016/j.carbpol.2016.05.095

[60] Betancor, L. et al. Different mechanisms of protein immobilization on glutaraldehyde activated supports: effect of support activation and immobilization conditions. *Enzyme Microb. Technol.* **39**, 4,877–882. 10.1016/j.enzmictec.2006.01.014 (2006).

[61] Mohamed, T. M. et al. Immobilization and characterization of Inulinase from Ulocladium atrum on nonwoven fabrics. *J. Biosci.* **39** (5), 785–793. 10.1007/s12038-014-9477-1 (2014). 25431408 10.1007/s12038-014-9477-1

[62] Verma, M. L., Rao, N. M., Tsuzuki, T., Barrow, C. J. & Puri, M. Suitability of Recombinant lipase immobilised on functionalised magnetic nanoparticles for fish oil hydrolysis. *Catalysts* **9** (5,420). 10.3390/catal9050420 (2019).

[63] Koh, A. L. & Sinclair, R. TEM Observations of Bio-Conjugated Streptavidin-Gold Nanoparticles, *MRS Online Proc. Libr.*, 1019,1,501, (2011). 10.1557/PROC-1019-FF05-01

[64] Verma, M. L., Naebe, M., Barrow, C. J. & Puri, M. Enzyme immobilisation on Amino-Functionalised Multi-Walled carbon nanotubes: structural and biocatalytic characterisation. *PLOS ONE*. **8** (9,e73642). 10.1371/journal.pone.0073642 (2013). 10.1371/journal.pone.0073642 PMC3772012 24069216

[65] Mayordomo, I., Randez-Gil, F. & Prieto, J. A. Isolation, Purification, and characterization of a Cold-Active lipase from *Aspergillus Nidulans*. *J. Agric. Food Chem.* **48** (1), 105–109. 10.1021/jf9903354 (2000). 10637060 10.1021/jf9903354

[66] Matuoog, N., Li, K. & Yan, Y. *Thermomyces lanuginosus* lipase immobilized on magnetic nanoparticles and its application in the hydrolysis of fish oil. *J. Food Biochem.* **42** (5,e12549). 10.1111/jfbc.12549 (2018).

[67] Hu, B., Pan, J., Yu, H. L., Liu, J. W. & Xu, J. H. Immobilization of Serratia marcescens lipase onto amino-functionalized magnetic nanoparticles for repeated use in enzymatic synthesis of diltiazem intermediate. *Process. Biochem.* **44** (9), 1019–1024. 10.1016/j.procbio.2009.05.001 (2009).

[68] Pavlidis, I. V. et al. Development of effective nanobiocatalytic systems through the immobilization of hydrolases on functionalized carbon-based nanomaterials. *Biocatalysis* **115**, 164–171. 10.1016/j.biortech.2011.11.007 (2012). 10.1016/j.biortech.2011.11.007 22113071

[69] Aghamolaei, M. et al. Preparation and characterization of stable core/ shell Fe3O4@Au decorated with an amine group for immobilization of lipase by covalent attachment. *RSC Adv.* **12** (10), 5971–5977. 10.1039/D1RA08147K (2022). 35424559 10.1039/d1ra08147k PMC8982027

[70] Ismail, M. et al. Pollution, toxicity and carcinogenicity of organic dyes and their catalytic bio-remediation. *Curr. Pharm. Des.* **25**, 34,3645–3663. 10.2174/1381612825666191021142026 (2019). 31656147 10.2174/1381612825666191021142026

[71] Parapat, R. Y. et al. Eco-friendly nanocatalysts: unleashing non-precious metal potential for methylene blue remediation. *E3S Web Conf.* **484** (03004). 10.1051/e3sconf/202448403004 (2024).

[72] Yao, L. W. et al. Insight into immobilization efficiency of lipase enzyme as a biocatalyst on the graphene oxide for adsorption of Azo dyes from industrial wastewater effluent. *J. Mol. Liq*. **354** (118849). 10.1016/j.molliq.2022.118849 (2022).

[73] Gulzar, T. et al. Bioremediation of synthetic and industrial effluents by Aspergillus Niger isolated from contaminated soil following a sequential strategy. *Molecules* **22** (12). 10.3390/molecules22122244 (2017). 10.3390/molecules22122244 PMC6149775 29258168

[74] Wang, J. et al. Biodegradation of Azo dyes by genetically engineered Azoreductase. *J. Environ. Sci. China*. **17**, 545–550 (2005). 16158576

[75] Mahmoud, M. E., El-Sharkawy, R. M. & Ibrahim, G. A. A. A novel Bionanocomposite from doped lipase enzyme into magnetic graphene oxide-immobilized-cellulose for efficient removal of methylene blue and malachite green dyes. *J. Mol. Liq*. **368** (120676). 10.1016/j.molliq.2022.120676 (2022).

[76] Goswami, D., Basu, J. K. & De, S. Lipase applications in oil hydrolysis with a case study on castor oil: a review. *Crit. Rev. Biotechnol.* **33** (1), 81–96. 10.3109/07388551.2012.672319 (2013). 22676042 10.3109/07388551.2012.672319

