# Supplementary material for: Synthesis of an enhanced nanobiocatalyst system from Aspergillus niger as single green source
Source: Sci Rep. 2025 Dec 23;15:44411. doi: 10.1038/s41598-025-31186-9 (PMC12738789; doi:10.1038/s41598-025-31186-9)
Supplement: Supplementary file 1 — Supplementary Material 1 [file 41598_2025_31186_MOESM1_ESM.docx]

**Synthesis of an enhanced nanobiocatalyst system from *Aspergillus niger* as single green source**

**Mohamed G. Radwan^1^, Tarek M. Mohamed^2^, Radwa H. Abou-Saleh^3,4*^, Hamed A. Abosharaf ^2*^**

^1^Health Sector, Faculty of Science, Galala University, Suez 43511, Egypt.

^2^Biochemistry Division, Chemistry Department, Faculty of Science, Tanta University, Tanta 31527, Egypt.

^3^Nanoscience and technology, Physics Department, Faculty of Science, Galala University, Suez 43511, Egypt.

^4^Biophysics division, Physics Department, Faculty of Science, Mansoura University, Mansoura 35516, Egypt.

**^*^Corresponding author**s:

**E-mail**: [**r.h.saleh@gu.edu.eg**](mailto:r.h.saleh@gu.edu.eg) - [**hamed_biochemistry@science.tanta.edu.eg**](mailto:hamed_biochemistry@science.tanta.edu.eg)

**Supplementary Table S1: Comparison of Conventional Multi-Source Nanobiocatalyst Systems with the Proposed Single-Source Approach.**

| **Reference** | **Enzyme Source (Organism)** | **Nanoparticle Support** | **Synthesis Method of Support** | **Source Integration** |
| --- | --- | --- | --- | --- |
| [1] | *Thermomyces lanuginosus* Lipase | Superparamagnetic iron oxide nanoparticles | Co-precipitation | Separate Sources |
| [2] | *Candida antarctica* lipase B (CALB) | Iron oxide magnetic nanoparticles (Fe₃O₄) | Co-precipitation | Separate Sources |
| [3] | *Candida rugosa* lipase | Fe₃O₄ superparamagnetic nanoparticles | Co-precipitation | Separate Sources |
| [4] | *Candida antarctica* lipase B (Lipozyme®) | Iron oxide nanoparticles | Co-precipitation | Separate Sources |
| [5] | Porcine pancreatic lipase (PPL) | Ag/IONPs | Plant Extract & Co-precipitation | Separate Sources |
| [6] | *Burkholderia sp.* lipase | Ferric silica nanocomposite | Commercial IONPs + Silica Coating | Separate Sources |
| **This Study** | *Aspergillus niger* Lipase | Iron Oxide Nanoparticles | Biosynthesis (from *A. niger*) | Single Source |


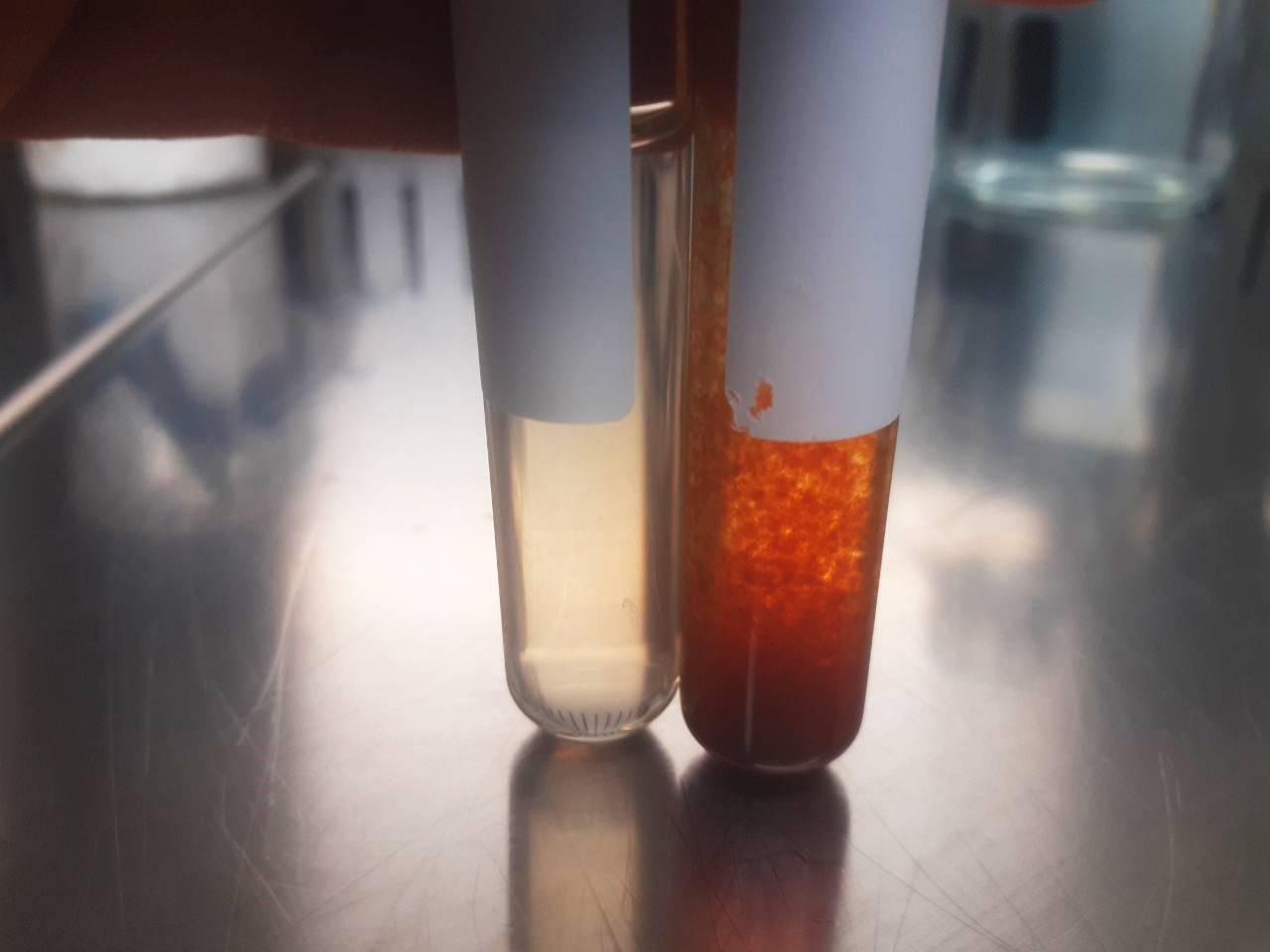


**b**

**a**

**Fig. S1. Biosynthesis of IONPs using the cell free filtrate of A. niger ATCC 16878. bottle (a) is a control contains only fungal filtrate without salt, and bottle (b) is a test.**

**Fig. S2. UV‒Visible spectra of IONPs**


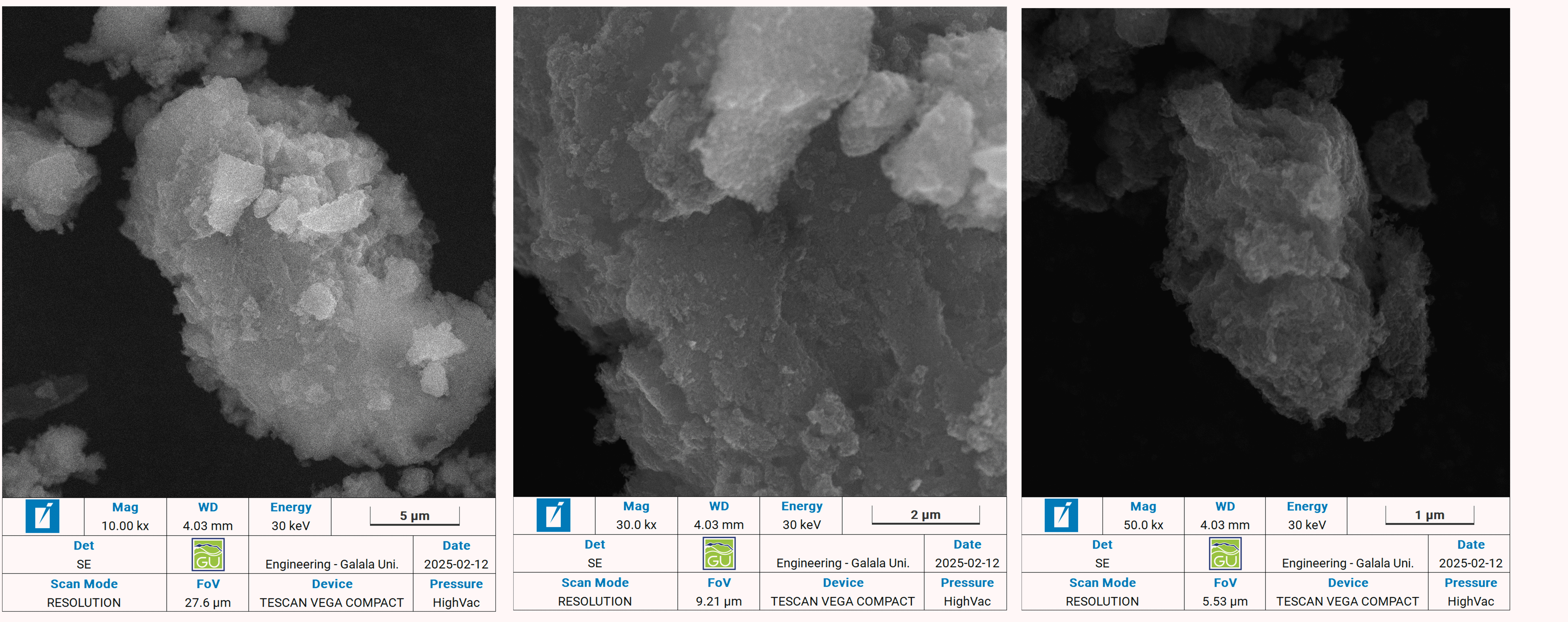


**Fig. S.3 | Multi-scale SEM analysis of biosynthesized IONPs. Micrographs of the iron oxide nanoparticles are shown at increasing magnifications: (a) 10,000x, (b) 30,000x, and (c) 50,000x. The lower magnification images (a, b) reveal the tendency of the primary nanoparticles to form larger, micron-scale aggregates. The higher magnification image (c) provides a clearer view of the structure within these aggregates, showing the constituent primary nanoparticles.**

**c**

**b**

**a**

**
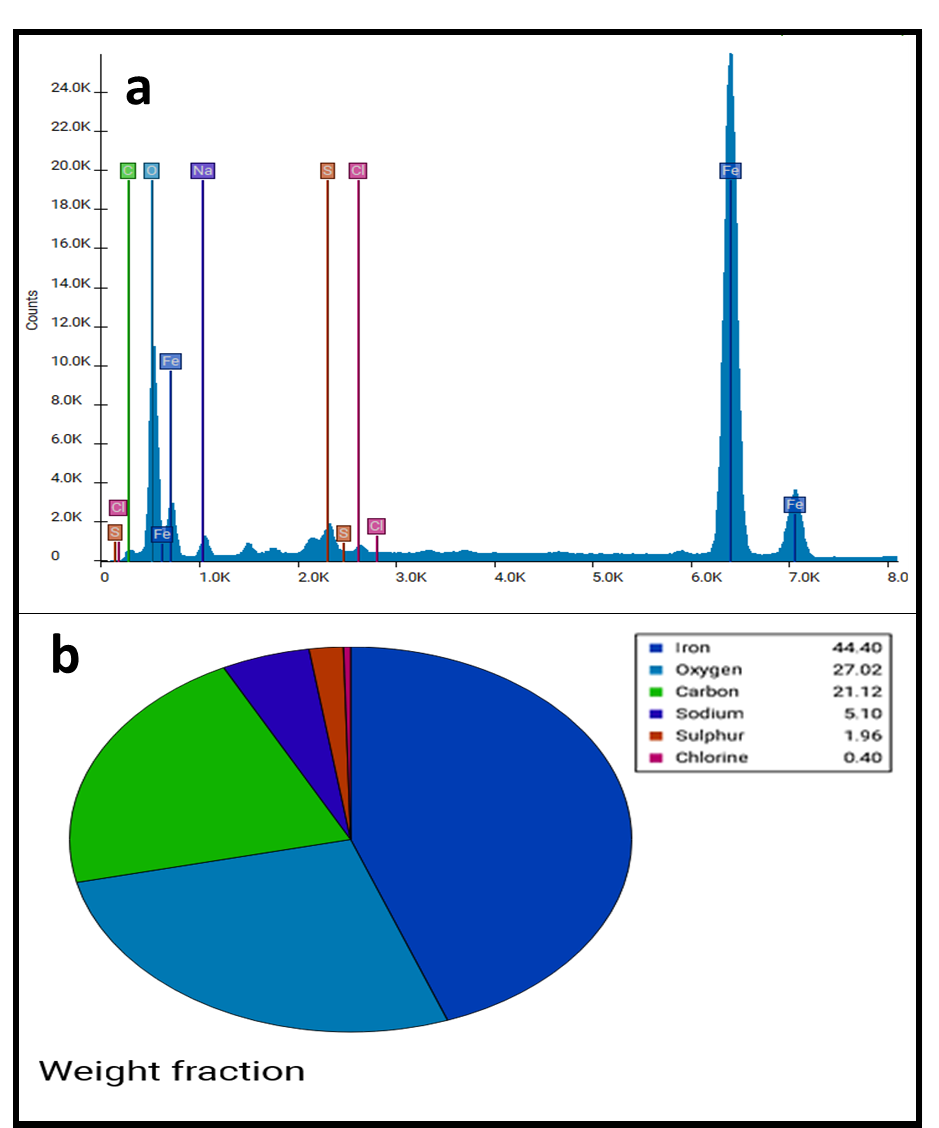
**

**Fig. S4. (a) EDX spectrum of the biosynthesized IONPs. (b)** **Charting of the weight fraction**


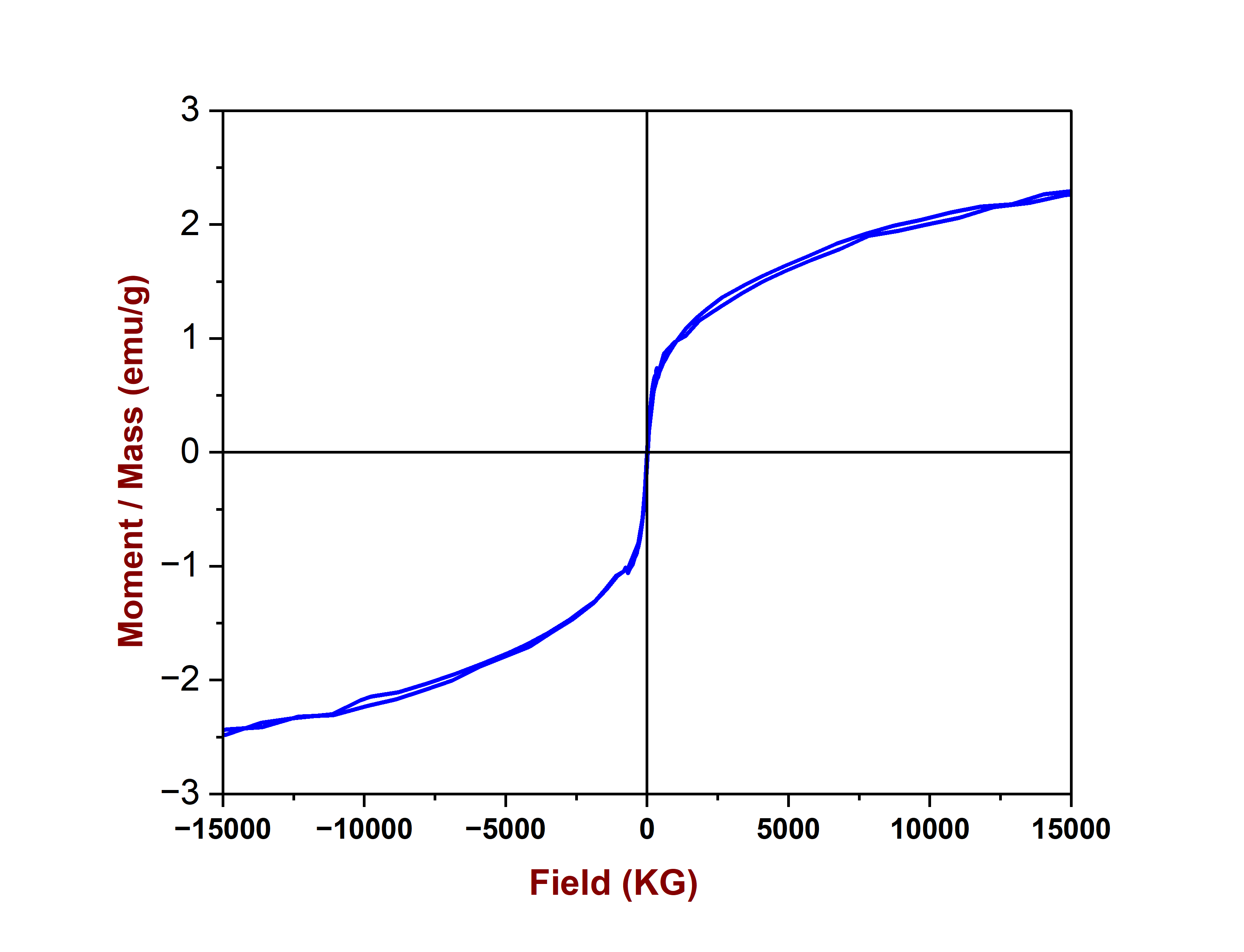


**Fig. S5. VSM analysis of biosynthesized IONPs**


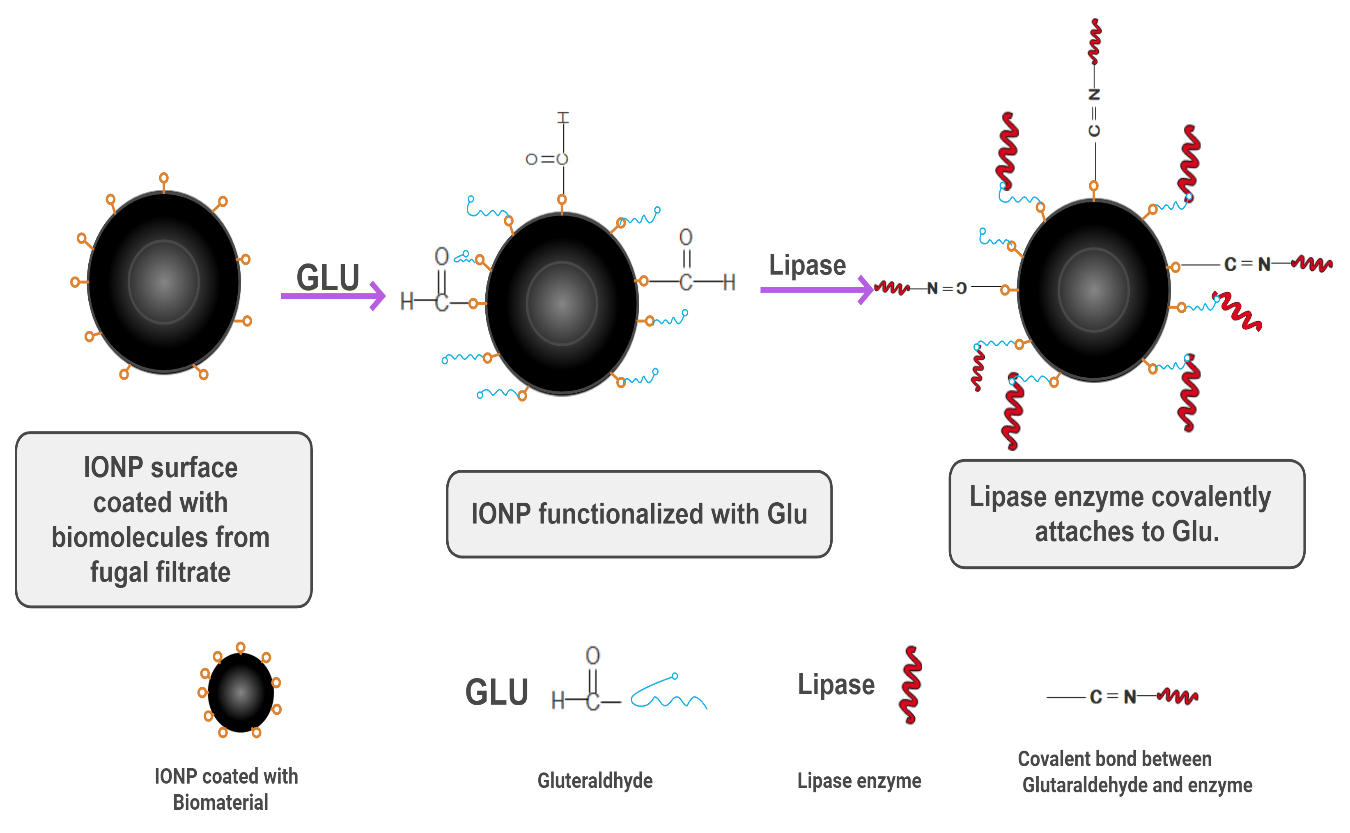


**Fig. S6. Schematic representation of covalent immobilization between lipase and surface functionalized IONPs**





**Fig. S7. Shelf-life stability of free and immobilized lipase at different storage temperatures (25,4°C)**


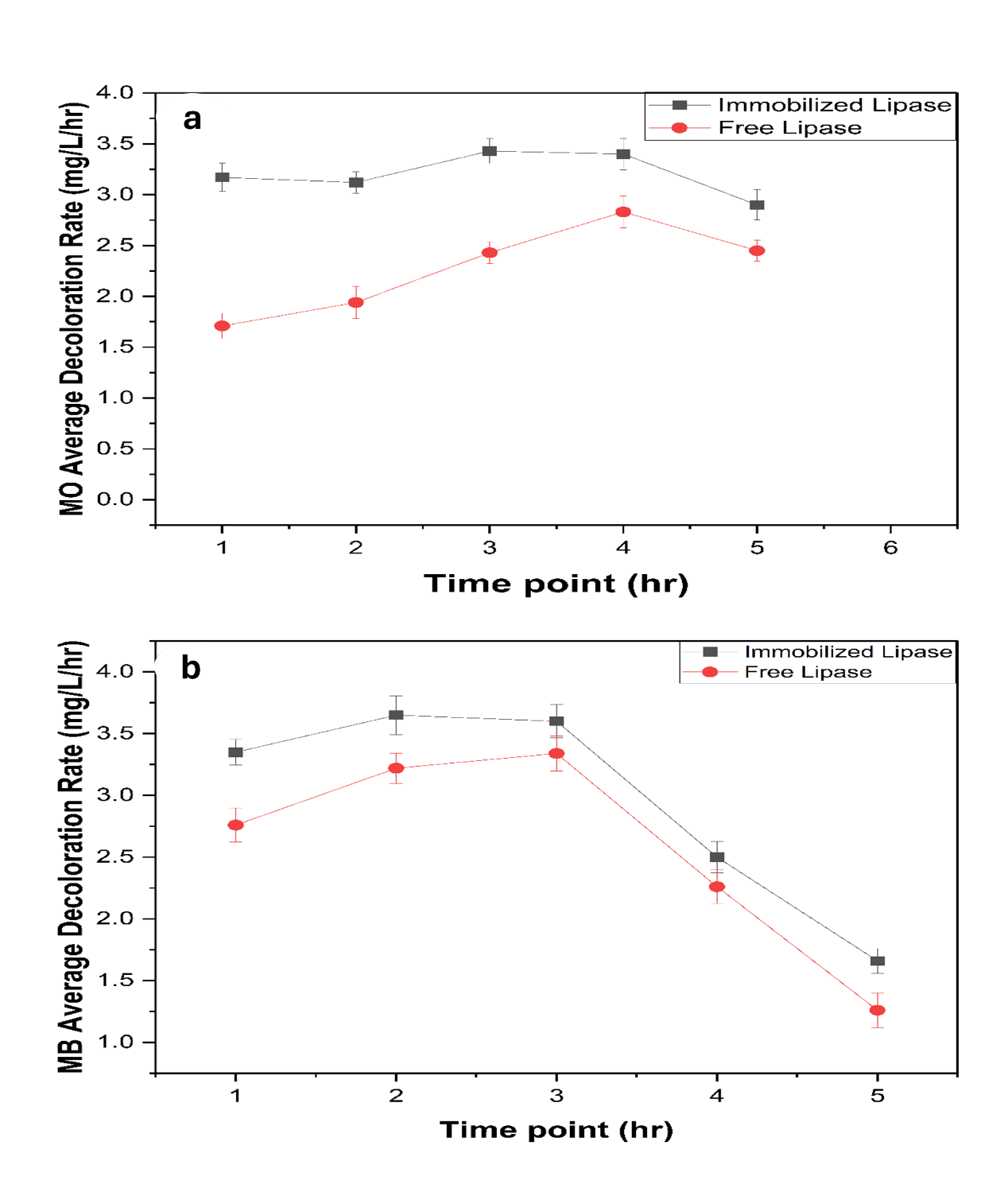
**Fig. S8. (A) Methyl orange (MO) and (B) methylene blue (MB) degradation rates in terms of the average deceleration rate over time. Data represent the means ± SDs (n = 3).**

# References

[1] M. Henrique da Silva Cavalcanti *et al.*, “Immobilization of Thermomyces lanuginosus lipase via ionic adsorption on superparamagnetic iron oxide nanoparticles: Facile synthesis and improved catalytic performance,” *Chem. Eng. J.*, vol. 431, p. 134128, Mar. 2022, doi: 10.1016/j.cej.2021.134128.

[2] V. M. Costa, M. C. M. D. Souza, P. B. A. Fechine, A. C. Macedo, and L. R. B. Gonçalves, “NANOBIOCATALYTIC SYSTEMS BASED ON LIPASE-Fe3O4 AND CONVENTIONAL SYSTEMS FOR ISONIAZID SYNTHESIS: A COMPARATIVE STUDY,” *Braz. J. Chem. Eng.*, vol. 33, no. 3, pp. 661–673, Sep. 2016, doi: 10.1590/0104-6632.20160333s20150137.

[3] K. Solanki and M. N. Gupta, “Simultaneous purification and immobilization of Candida rugosa lipase on superparamagnetic Fe3O4 nanoparticles for catalyzing transesterification reactions,” *New J. Chem.*, vol. 35, no. 11, p. 2551, 2011, doi: 10.1039/c1nj20277d.

[4] M. Viñambres, M. Filice, and M. Marciello, “Modulation of the Catalytic Properties of Lipase B from Candida antarctica by Immobilization on Tailor-Made Magnetic Iron Oxide Nanoparticles: The Key Role of Nanocarrier Surface Engineering,” *Polymers*, vol. 10, no. 6, p. 615, Jun. 2018, doi: 10.3390/polym10060615.

[5] R. Konwarh, M. Shail, T. Medhi, M. Mandal, and N. Karak, “Sonication assisted assemblage of exotic polymer supported nanostructured bio-hybrid system and prospective application,” *Ultrason. Sonochem.*, vol. 21, no. 2, pp. 634–642, Mar. 2014, doi: 10.1016/j.ultsonch.2013.10.014.

[6] D.-T. Tran, C.-L. Chen, and J.-S. Chang, “Immobilization of Burkholderia sp. lipase on a ferric silica nanocomposite for biodiesel production,” *J. Biotechnol.*, vol. 158, no. 3, pp. 112–119, Apr. 2012, doi: 10.1016/j.jbiotec.2012.01.018.
